# Integrated Phenotypic and Transcriptomic Profiling Positions ONC212 as a Lead Imipridone in Androgen-Independent Prostate Cancer Models

**DOI:** 10.3390/ijms27104597

**Published:** 2026-05-20

**Authors:** Fatima Ghamlouche, Amani Yehya, Abdallah Kurdi, Sana Hachem, Varun V. Prabhu, Georges Daoud, Wassim Abou-Kheir

**Affiliations:** 1 Department of Anatomy, Cell Biology and Physiological Sciences, Faculty of Medicine, American University of Beirut, Beirut P.O. Box 11-0236, Lebanon; 2 Department of Biochemistry and Molecular Genetics, Faculty of Medicine, American University of Beirut, Beirut P.O. Box 11-0236, Lebanon; 3 Chimerix, Inc., Durham, NC 27713, USA

**Keywords:** prostate cancer, prostate cancer stem cells, imipridones, ONC206, ONC212, 3D culture, transcriptomics, integrated stress response, unfolded protein response

## Abstract

Prostate cancer (PCa) remains lethal at advanced stages, partly due to stem-like subpopulations known as prostate cancer stem cells (PCSCs) that sustain tumor growth and therapeutic resistance. Imipridones are small-molecule anticancer agents, with next-generation derivatives ONC206 and ONC212 designed for enhanced potency and broader activity. This study compared their antitumor efficacy and mechanisms in advanced androgen-independent PCa (AIPC) models, namely DU145 and PC3 cells, using two- and three-dimensional systems encompassing bulk cancer cells and PCSCs. DU145 and PC3 AIPC cells were treated with ONC201 (parent compound), ONC206, or ONC212. Functional assays assessed proliferation, viability, migration, invasion, PCa spheroids formation, cell cycle distribution, and mitochondrial membrane potential and mass, while RNA sequencing defined transcriptional responses. ONC212 was the most potent derivative, inhibiting proliferation and migration and abolishing PCa spheroids at nanomolar doses, whereas ONC201 and ONC206 required higher concentrations. Transcriptomic analyses revealed shared repression of DNA replication and cell-cycle transition programs, with activation of integrated stress and unfolded protein responses (ISR/UPR) and FOXO signaling. ONC206 favored PERK–ATF4-mediated apoptosis with reduced DNA repair, while ONC212 more strongly impacted oxidative phosphorylation-related pathways and mitochondrial RNA processing. Imipridones induced a time-dependent cell-cycle redistribution with increased sub-G1 accumulation and modulated mitochondrial membrane potential and mass in a context-dependent manner. Collectively, these findings position ONC212 as a leading imipridone candidate in AIPC models, combining potent inhibition of tumor and stem-like cell functions with a coherent stress-response signature that supports further translational evaluation.

## 1. Introduction

Prostate cancer (PCa) is a leading malignancy among men worldwide, ranking second in incidence and fifth in cancer-related mortality. According to GLOBOCAN 2022 data, PCa is the most frequently diagnosed cancer and the second leading cause of cancer mortality in men in 118 and 52 countries, respectively [1]. Clinically, PCa spans a broad spectrum ranging from indolent, localized tumors to aggressive, treatment-refractory metastatic disease. Although most cases are detected at an organ-confined stage, prognosis varies widely among patients. This variability is largely driven by the substantial spatial, histomorphological, genetic, and molecular heterogeneity of PCa—both between patients and within individual tumors—which poses major challenges for accurate diagnosis, effective clinical management, and the development of durable therapeutic strategies [2,3]. A growing body of evidence implicates prostate cancer stem cells (PCSCs) as central contributors to this heterogeneity. PCSCs are slow-cycling, tumor-initiating cells with self-renewal and pluripotency capacities, enabling tumor maintenance and progression while conferring therapeutic resistance and promoting recurrence [4,5].

Clinically, PCa lethality is largely associated with progression to advanced, treatment-refractory disease states. Although many patients respond initially to androgen deprivation therapy and androgen receptor signaling inhibitors —and, where used, chemotherapy—a substantial proportion ultimately develop incurable metastatic, castration-resistant PCa (mCRPC) [6,7]. Within advanced mCRPC, a clinically important subset sustains tumor growth despite castrate androgen levels while exhibiting reduced reliance on canonical androgen receptor (AR) signaling; this phenotype is often referred to as androgen-independent/AR-indifferent disease [8]. During progression, PCa undergoes dynamic metabolic rewiring, including increased mitochondrial bioenergetic activity, which has been linked to therapeutic resistance [9]. Since treatment-refractory and stem-like tumor subpopulations can exhibit metabolic plasticity and frequently rely on mitochondrial programs to sustain survival and self-renewal [10,11], targeting mitochondrial/metabolic dependencies may represent a therapeutic strategy to manage advanced androgen-independent PCa (AIPC) and resilient PCSC subpopulations.

Imipridones have emerged as a promising class of small-molecule compounds with demonstrated anticancer activity across diverse solid and hematologic malignancies. These agents share a tri-heterocyclic imidazopyridopyrimidone scaffold, with pharmacological properties modulated by distinct substitutions on the peripheral benzyl rings [12,13,14]. Imipridones engage multiple intracellular targets that may vary with respect to the chemical structure. They may stimulate the p53-independent tumor necrosis factor-related apoptosis-inducing ligand (TRAIL) signaling. Specifically, imipridones may increase the transcription of TRAIL, which will bind to its death receptor DR4/5 (TRAIL-R1/2) on the cell membrane, leading to a receptor-ligand complex that prompts the natural immune response against tumor cells, ultimately leading to cancer cell apoptosis while sparing normal host cells. Imipridones may also induce an early-stage integrated stress response (ISR) and consequently activate the ATF4/CHOP/DR5 axis, generally triggering apoptosis. Moreover, imipridones may antagonize G protein-coupled receptors (GPCRs), including the dopamine receptor D2 (DRD2), which is overexpressed in a broad range of cancers, and the orphan GPCR GPR132, which acts as a tumor suppressor. Recent studies report that their activity can involve disruption of mitochondrial metabolism. Specifically, imipridones can act as an allosteric agonist and hyperactivate the mitochondrial ATP-dependent caseinolytic protease P (ClpP). Consequently, the imipridone-ClpP complex promotes degradation of key mitochondrial proteins, including electron transport chain (ETC) components, thereby impairing oxidative phosphorylation (OXPHOS) and mitochondrial energy production in cancer cells and engaging downstream stress pathways that can contribute to tumor cell death [13,14,15,16,17].

**Figure 1:**
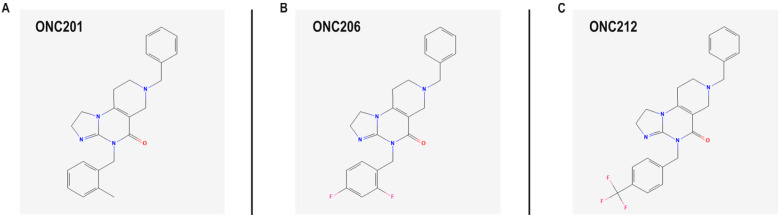
Chemical structures of the first-in-class imipridone, ONC201, and its two derivatives, ONC206 and ONC212. (**A**) ONC201: 11-benzyl-7-[(2-methylphenyl)methyl]-2,5,7,11-tetrazatricyclo[7.4.0.0^2^,^6^]trideca-1(9),5-dien-8-one. (**B**) ONC206: 11-benzyl-7-[(2,4-difluorophenyl)methyl]-2,5,7,11-tetrazatricyclo[7.4.0.0^2^,^6^]trideca-1(9),5-dien-8-one. (**C**) ONC212: 11-benzyl-7-[[4-(trifluoromethyl)phenyl]methyl]-2,5,7,11-tetrazatricyclo[7.4.0.0^2^,^6^]trideca-1(9),5-dien-8-one. The two-dimensional chemical structures were adopted from [19,20,21].

The first-in-class imipridone ONC201 (dordaviprone) (Figure 1A; 11-benzyl-7-[(2-methylphenyl)methyl]-2,5,7,11-tetrazatricyclo[7.4.0.0^2^,^6^]trideca-1(9),5-dien-8-one) has shown broad-spectrum antitumor activity in preclinical and clinical contexts across solid and hematologic malignancies, including PCa [18,19]. Next-generation derivatives were subsequently developed to achieve greater potency and expanded mechanistic profiles [12,13,14]. ONC206 (Figure 1B; 11-benzyl-7-[(2,4-difluorophenyl)methyl]-2,5,7,11-tetrazatricyclo[7.4.0.0^2^,^6^]trideca-1(9),5-dien-8-one) was derived from ONC201 by substituting the 7-position arylmethyl group from 2-methylbenzyl to 2,4-difluorobenzyl. On the other hand, ONC212 (Figure 1C; 11-benzyl-7-[[4-(trifluoromethyl)phenyl]methyl]-2,5,7,11-tetrazatricyclo[7.4.0.0^2^,^6^]trideca-1(9),5-dien-8-one) was derived from ONC201 by replacing the same 7-position arylmethyl group with 4-(trifluoromethyl)benzyl [12,20,21].

Both ONC206 and ONC212 have demonstrated superior preclinical efficacy across diverse malignancies, including central nervous system (CNS), ovarian, endometrial, pancreatic, colorectal, and hematologic cancers [13,22,23,24,25,26,27,28,29]. ONC206 exhibited nanomolar antineoplastic activity and antimetastatic potential and is under evaluation in early-phase clinical trials in patients with CNS tumors (NCT04541082; NCT04732065). ONC212 similarly showed robust antitumor effects at low nanomolar concentrations, with favorable pharmacokinetic and safety profiles, and consistently outperformed ONC201 in several preclinical models [13,30]. Collectively, these findings establish ONC206 and ONC212 as promising next-generation imipridones with enhanced biological activity and translational potential. However, their comparative efficacy and underlying mechanisms in PCa, particularly in advanced androgen-independent contexts, remain to be defined.

To address these gaps, we systematically compared ONC206 and ONC212 with ONC201 in advanced human AIPC models, examining effects on both the bulk tumor population and stem/progenitor-like cells using two-dimensional (2D) and three-dimensional (3D) culture systems. We integrated proliferation, migration, PCa spheroids’ formation, and cell-cycle analyses with assessments of mitochondrial membrane potential and mass, together with transcriptomic and pathway profiling to define shared and compound-specific mechanisms of action. This integrative approach provides a comparative view of therapeutic efficacy and identifies coordinated stress-response and mitochondrial regulatory changes that warrant further mechanistic validation.

## 2. Results

### 2.1. ONC206 or ONC212 Reduced the Proliferation and Viability of Human AIPC Cells, with ONC212 Being the Most Potent

**Figure 2:**
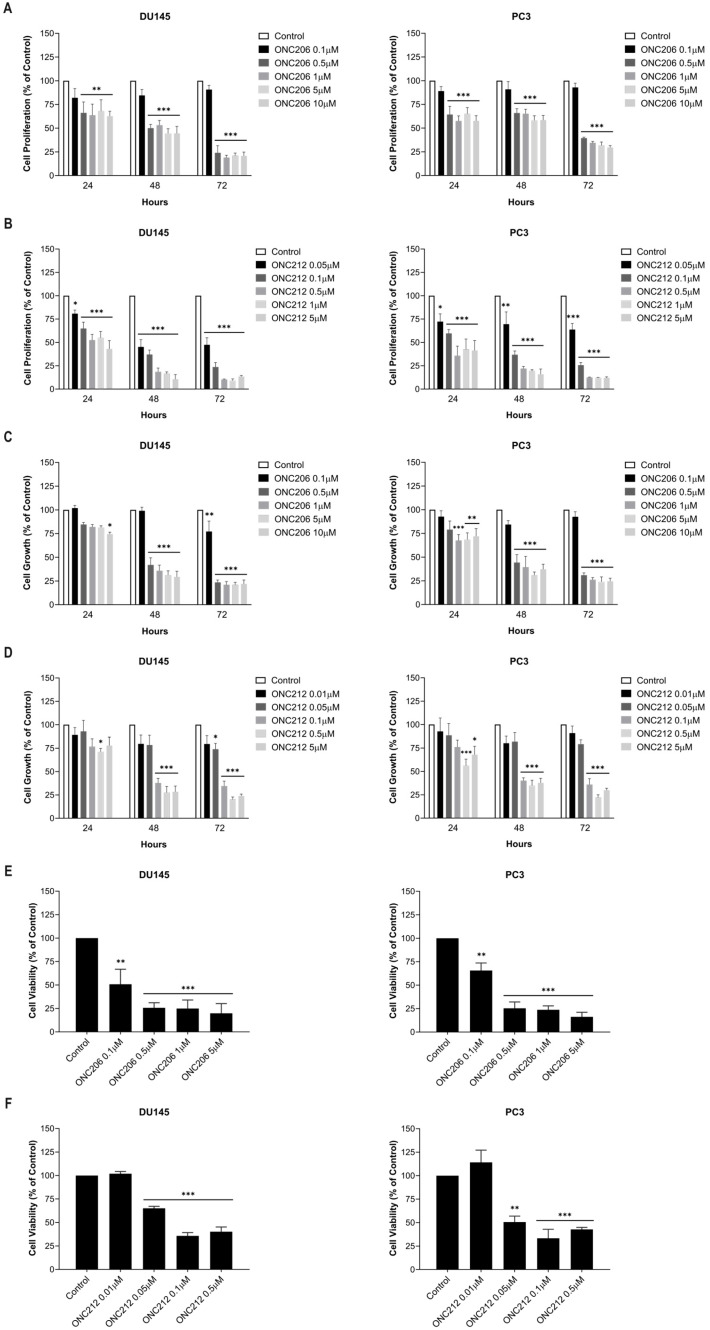
ONC206 or ONC212 reduced the proliferation, growth, and viability of human androgen-independent prostate cancer cells in a time- and dose-dependent manner. DU145 and PC3 cells were treated for 24, 48, or 72 h with the indicated concentrations of ONC206 (**A**) or ONC212 (**B**), and cell proliferation was measured by thiazolyl blue tetrazolium bromide (MTT) assay. DU145 and PC3 cells were treated for 24, 48, or 72 h with the indicated concentrations of ONC206 (**C**) or ONC212 (**D**), and cell growth was measured by Sulforhodamine B (SRB) assay. DU145 and PC3 cells were treated for 48 h with the indicated concentrations of ONC206 (**E**) or ONC212 (**F**), and cell viability was measured by trypan blue exclusion assay. Results are normalized to the vehicle control at each time point and shown as mean ± SEM from ≥ 3 independent experiments. Statistical significance was assessed by two-way ANOVA with Tukey’s or Dunnett’s post hoc multiple-comparisons tests; * *p* < 0.05, ** *p* < 0.01, *** *p* < 0.001.

As a preliminary test, the antiproliferative effect of the imipridones on PCa cells was first evaluated using the thiazolyl blue tetrazolium bromide (MTT) assay. Two well-established AIPC cell lines, DU145 and PC3, were treated with increasing concentrations of ONC201 (0.1–10 µM), ONC206 (0.1–10 µM), or ONC212 (0.05–5 µM) for up to 72 h. All three compounds significantly reduced proliferation in a time- and dose-dependent manner in PCa cells, with inhibitory effects detected at 48 h starting from 5 µM for ONC201 (*p* = 0.0004 for DU145; *p* = 0.0124 for PC3) (Supplementary Figure S1), 0.5 µM for ONC206 (*p* < 0.0001 for both cell lines) (Figure 2A), and 0.05 µM for ONC212 (*p* < 0.0001 for DU145; *p* = 0.0052 for PC3) (Figure 2B). Notably, ONC212 was the most potent, reducing proliferation by more than two-fold compared to ONC201 and ONC206 at 0.1 µM after 48 h (DU145: *p* = 0.0003 vs. ONC201, *p* = 0.0006 vs. ONC206; PC3: *p* < 0.0001 vs. ONC201, *p* = 0.0002 vs. ONC206) (Supplementary Figure S2).

Since imipridones may exert their effects by disrupting mitochondrial metabolism, a Sulforhodamine B (SRB) assay was used to confirm growth inhibition and determine the Growth Inhibitory Concentration 50% (GI_50_). This assay quantifies total cellular protein content and is independent of mitochondrial metabolic function. Additionally, to improve the generalizability of our findings, we assessed the anti-proliferative effects of imipridones in the 22Rv1 cell line, which largely has an androgen-independent growth ability. All three compounds significantly inhibited PCa cell growth in a time- and dose-dependent manner. At 48 h post-treatment, significant growth inhibition was observed at 5 µM for ONC201 (*p* < 0.0001 for all three cell lines) (Supplementary Figure S3A), 0.5 µM for ONC206 (*p* < 0.0001 for all three cell lines) (Figure 2C, Supplementary Figure S3B), and 0.1 µM for ONC212 (*p* < 0.0001 for all three cell lines) (Figure 2D, Supplementary Figure S3C). Consistent with the MTT results, ONC212 was the most potent, leading to at least two-fold reduction in DU145 and PC3 cell growth compared to ONC201 or ONC206 at 0.1 µM after 48 h (*p* < 0.0001 for both cell lines vs. ONC201 or ONC206) (Supplementary Figure S4A). In fact, 0.1 µM ONC212 achieved comparable growth inhibition to 5 µM ONC201 and 0.5 µM ONC206 after 48 h in both cell lines, concentrations that approximately corresponded to the mean GI_50_ values and were used in subsequent assays (Supplementary Figures S4B and Table S1).

In addition, the effect of ONC201, ONC206, and ONC212 on AIPC cell viability was assessed using the trypan blue exclusion assay, focusing on 48 h post-treatment. All three imipridones significantly inhibited DU145, PC3, and 22Rv1 cell viability (Figure 2E,F, Supplementary Figure S5). Similarly to the MTT and SRB assays, ONC212 demonstrated higher potency compared to ONC201 and ONC206 in the three tested cell lines (Figure 2E,F, Supplementary Figure S5).

Collectively, these results demonstrate that ONC212 is the most potent imipridone in reducing proliferation, growth, and viability of AIPC cells, achieving significant effects at markedly lower concentrations than ONC201 and ONC206.

### 2.2. ONC212 Reduced the Migratory Capacity of Human AIPC Cells

**Figure 3:**
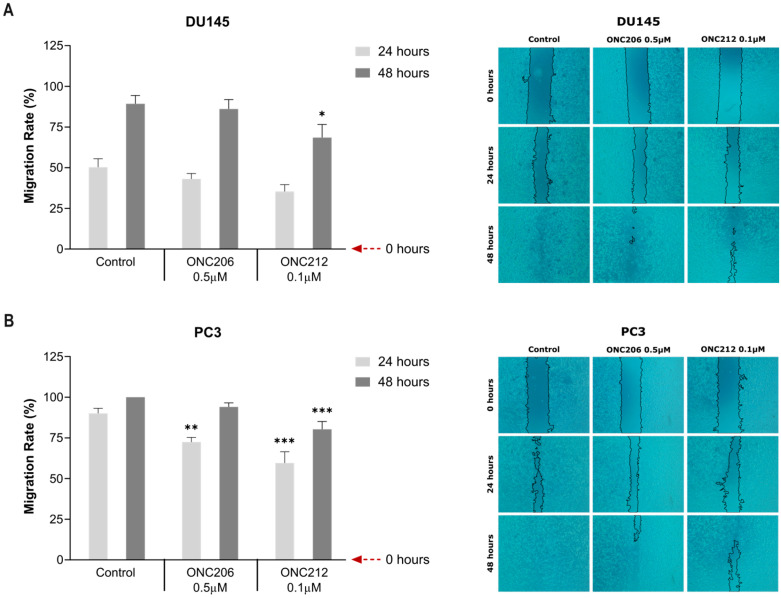
ONC212 reduced the migratory capacity of human androgen-independent prostate cancer cells. Confluent monolayers of DU145 (**A**) and PC3 (**B**) cells were pretreated with mitomycin C to inhibit proliferation, scratched with a sterile 200 µL pipette tip, and treated with ONC206 (0.5 µM) or ONC212 (0.1 µM); vehicle served as control. Images were acquired at 0, 24, and 48 h (5× magnification). Wound area was quantified using ImageJ, and migration rate (%) was calculated and presented. Bars show mean ± SEM from ≥4 independent experiments. Statistical significance was assessed by two-way ANOVA with Dunnett’s post hoc test vs. time-matched control; * *p* < 0.05, ** *p* < 0.01, *** *p* < 0.001.

To assess the effects of imipridones on cell migration in AIPC models, the wound-healing assay was performed. In DU145 cells, treatment with the GI_50_ concentration of ONC212 (0.1 µM) significantly reduced the migration rate at 48 h compared with control (*p* = 0.0231) (Figure 3A). In PC3 cells, treatment with the corresponding GI_50_ concentrations of ONC201 (5 µM) (Supplementary Figure S6), ONC206 (0.5 µM) (Figure 3B), or ONC212 (0.1 µM) (Figure 3B) for 24 h significantly inhibited cell migration (*p* = 0.0034 for ONC201; *p* = 0.0014 for ONC206; *p* < 0.0001 for ONC212). Importantly, treatment with ONC212 (0.1 µM) for 48 h (Figure 3B) significantly inhibited PC3 cell migration (*p* = 0.0006).

ONC212 was the only compound that significantly reduced migration in both AIPC cell lines at 48 h, at which point untreated control wounds were nearly closed (Figure 3, Supplementary Figure S6). These findings indicate that ONC212 exerts the most potent and sustained anti-migratory activity in AIPC cells, achieving significant inhibition at nanomolar concentrations.

### 2.3. ONC206 or ONC212 Inhibited the Growth of Human AIPC Spheroids in 3D In Vitro Culture Systems, with ONC212 Being the Most Potent

**Figure 4:**
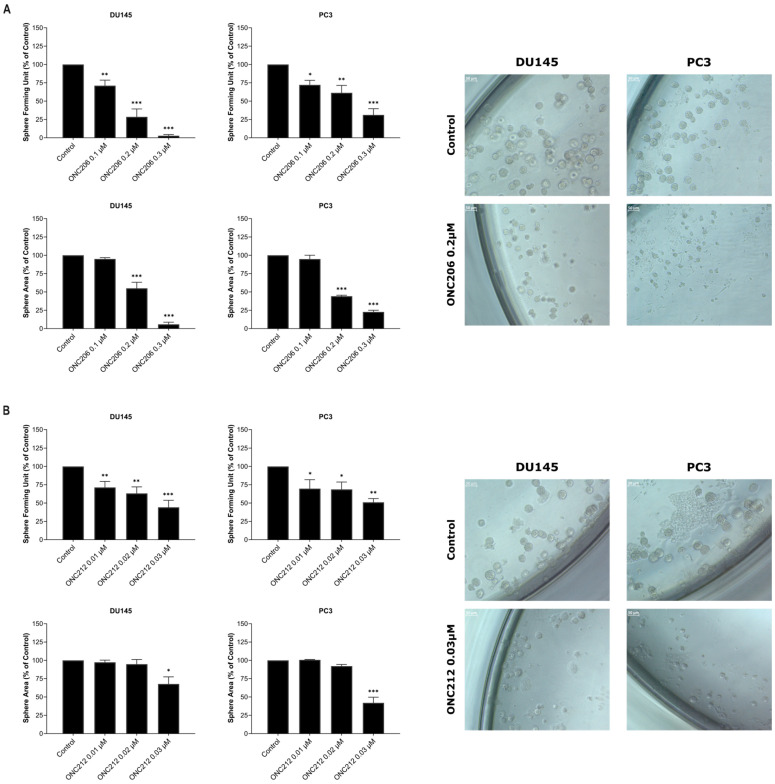
ONC206 or ONC212 reduced the formation and growth of human androgen-independent prostate cancer spheroids in three-dimensional culture. DU145 and PC3 cells were embedded in Matrigel™ and cultured with the indicated concentrations of ONC206 (**A**) or ONC212 (**B**), replenished every other day. Spheres were counted 7–8 days post-seeding and imaged using an Axiovert light microscope at 10× magnification. The number of spheres counted is expressed as sphere-forming unit (SFU %), normalized to the vehicle control. The mean sphere area (µm^2^) was determined from ≥30 spheres per condition. Data represents the mean ± SEM of ≥3 independent experiments. Statistical significance was assessed by one-way ANOVA with Holm-Šídák’s post hoc multiple-comparisons test; * *p* < 0.05, ** *p* < 0.01, *** *p* < 0.001. Representative images correspond to the quantitative SFU % and sphere area (scale = 50 µm).

The effects of imipridones on PCa spheroid-forming capacity of stem/progenitor-like subpopulations in PCa cells were assessed using a first-generation (G1) 3D sphere-formation assay. DU145 and PC3 cells embedded in Matrigel™ were treated with increasing concentrations of ONC201 (0.5–1.5 µM), ONC206 (0.1–0.3 µM), or ONC212 (0.01–0.03 µM), and sphere number and size were monitored over 7–8 days. Treatment with concentrations of 1.5 µM ONC201, 0.1 µM ONC206, or 0.01 µM ONC212 resulted in a significant reduction in sphere-forming unit (SFU) in both cell lines compared with controls (DU145: *p* = 0.0001 vs. ONC201, *p* = 0.0030 vs. ONC206, *p* = 0.0015 vs. ONC212; PC3: *p* = 0.0001 vs. ONC201, *p* = 0.0199 vs. ONC206, *p* = 0.0404 vs. ONC212) (Figure 4, Supplementary Figure S7). Effects on sphere formation were paralleled by reduced sphere growth. Treatment with concentrations of 1.5 µM ONC201, 0.2 µM ONC206, or 0.03 µM ONC212 significantly decreased average sphere area relative to controls in DU145 and PC3 cells (DU145: *p* < 0.0001 vs. ONC201, *p* < 0.0001 vs. ONC206, *p* = 0.0148 vs. ONC212; PC3: *p* = 0.0044 vs. ONC201, *p* < 0.0001 vs. ONC206, *p* < 0.0001 vs. ONC212) (Figure 4, Supplementary Figure S7). 

Overall, ONC212 suppressed PCa spheroid formation at ~50-fold and ~10-fold lower concentrations than ONC201 and ONC206, respectively, supporting enhanced activity against stem/progenitor-like subpopulations.

### 2.4. ONC206 or ONC212 Induced Transcriptomic Alterations in Human AIPC Cells and Engaged Overlapping and Distinct Enriched Signaling

**Figure 5:**
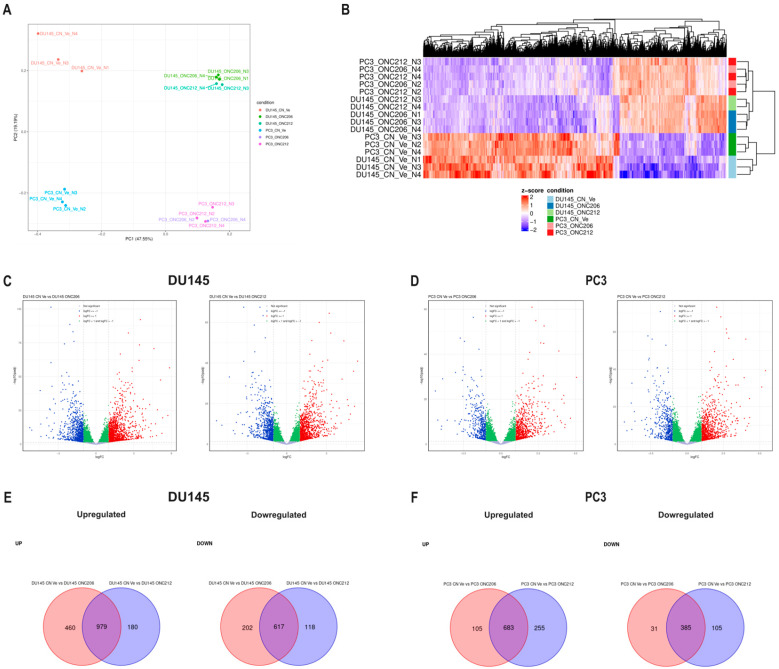
ONC206 or ONC212 reprogrammed gene expression in human androgen-independent prostate cancer cells. (**A**) Principal component analysis (PCA) of variance-stabilized RNA-sequencing counts showing sample clustering based on global transcriptional profiles of DU145 and PC3 cells treated with ONC206 (0.5 µM) or ONC212 (0.1 µM) for 48 h, relative to vehicle controls. PCA was performed using the top 500 most variable genes, with normalization and scaling applied to log_2_-transformed counts. (**B**) Hierarchical clustering heatmap of differentially expressed genes (DEGs) identified across all treatment conditions (│log_2_FC│≥ 1; *p* < 0.05). Expression values were z-score–normalized by gene across samples. Columns represent DEGs, and rows represent individual biological replicates. Color scale indicates relative expression intensity (red = upregulated, blue = downregulated). (**C**,**D**) Volcano plots of DEGs in DU145 (**C**) and PC3 (**D**) cells: vehicle vs. ONC206 (**left**) and vehicle vs. ONC212 (**right**). Red, upregulated (log_2_FC ≥ 1); blue, downregulated (log_2_FC ≤ −1); other points, not meeting the DEG threshold. (**E**,**F**) Venn diagrams showing overlap between ONC206- and ONC212-induced DEGs in DU145 (**E**) and PC3 (**F**) cells, for upregulated and downregulated genes; values indicate gene counts. CN Ve, control vehicle.

Given the distinct, yet enhanced, phenotypic potencies of ONC206 and ONC212, transcriptomic profiling by RNA-sequencing (RNA-seq) was conducted to identify differentially expressed genes (DEGs) and pathway alterations in PCa cells relative to untreated controls. Principal component analysis (PCA) revealed a pronounced separation between DU145 and PC3 samples, with untreated samples clustering apart along the second principal component, underscoring the inherent transcriptional heterogeneity between these AIPC cell lines (Figure 5A). Within each cell line, ONC206- or ONC212-treated samples clustered distinctly from their respective controls along the first principal component, indicating substantial drug-induced transcriptional reprogramming (Figure 5A). This separation was mirrored by hierarchical clustering of the top 50 variable genes, which showed discrete expression patterns between untreated controls and imipridone-treated samples and segregated DU145 and PC3 into discrete branches (Figure 5B).

When compared to their untreated counterparts, both ONC206 and ONC212 induced significant transcriptomic alterations in DU145 and PC3 cells, meeting the thresholds of an absolute log_2_ fold change ≥ 1 and adjusted *p*-value < 0.05 (Figure 5C,D, Supplementary Table S2). In DU145 cells, ONC206 differentially expressed 2258 genes (1439 downregulated and 819 upregulated relative to control), while ONC212 affected 1894 genes (1159 down and 735 up relative to control), with 979 downregulated and 617 upregulated genes shared between the two treatments (Figure 5C,E, Supplementary Table S2). In PC3 cells, ONC206 altered 1204 genes (788 down and 416 up relative to control), whereas ONC212 affected 1428 genes (938 down and 490 up relative to control), with 683 downregulated and 385 upregulated genes common to both treatments (Figure 5D,F, Supplementary Table S2).

These results indicate that, while ONC206 and ONC212 modulate overlapping transcriptional programs, each compound also drives compound-specific gene expression changes that may contribute to their differential biological effects.

Validation of the transcriptomic findings was performed by assessing the expression levels of selected genes in AIPC cells using reverse transcription quantitative real-time PCR. Consistent with the RNA-seq data, *CDKN2C* and *RRM2* genes were statistically downregulated in imipridone-treated DU145 and PC3 cells (Supplementary Figure S8A). Conversely, *PPP1R15A* and *ERN1* genes were statistically upregulated in both cell lines (Supplementary Figure S8B). These results confirm the reliability of the RNA-seq dataset, providing a solid basis for subsequent pathway enrichment analysis to uncover the broader biological programs modulated by ONC206 or ONC212.

To identify altered signaling programs in AIPC cells after treatment with the imipridone derivatives—and to distinguish shared from drug-specific effects—we assessed pathway enrichment using Enrichr and Reactome. EnrichR identified multiple deregulated pathways associated with altered gene expression in DU145 and PC3 cells following treatment with ONC206 or ONC212 (Supplementary Table S3). Both drugs showed significant enrichment of G2/M checkpoint–related gene sets among downregulated transcripts across DU145 and PC3 cell lines (DU145: *p* = 2.8 × 10^−8^ vs. ONC206, *p* = 7.43 × 10^−7^ vs. ONC212; PC3: *p* = 5.35 × 10^−4^ vs. ONC206, *p* = 0.019 vs. ONC212). Additionally, DNA repair pathways were preferentially enriched among downregulated transcripts in DU145 cells (significant for ONC212: *p* = 0.013; borderline for ONC206: *p* = 0.052) (Supplementary Table S2). On the other hand, among upregulated transcripts, both ONC206 and ONC212 showed significant enrichment of gene sets related to p53 signaling (DU145: *p* = 0.002 vs. ONC206, *p* = 0.002 vs. ONC212; PC3: *p* = 2.91 × 10^−4^ vs. ONC206, *p* = 0.005 vs. ONC212), apoptosis (DU145: *p* = 0.005 vs. ONC206, *p* = 0.034 vs. ONC212; PC3: *p* = 0.001 vs. ONC206, *p* = 0.012 vs. ONC212), and the unfolded protein response (UPR) (DU145: *p* = 0.029 vs. ONC206, *p* = 0.034 vs. ONC212; PC3: *p* = 0.003 vs. ONC206, *p* = 0.033 vs. ONC212) in DU145 and PC3 cells (Supplementary Table S3).

**Figure 6:**
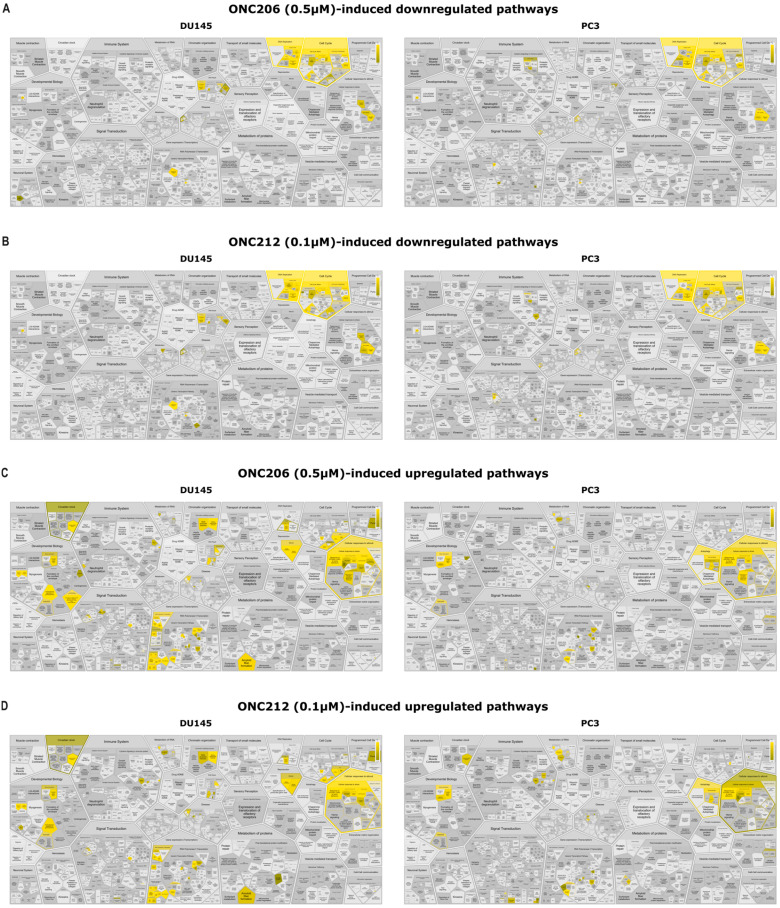
ONC206 or ONC212 enriched pathways related to cell-cycle programs suppression and activation of stress-adaptive pathways in human androgen-independent prostate cancer cells. (**A**,**B**) ReacFoam maps summarize pathway enrichment among downregulated transcripts after treatment with ONC206 (0.5 µM) (**A**) or ONC212 (0.1 µM) (**B**) in DU145 and PC3 cells. (**C**,**D**) ReacFoam maps summarize pathway enrichment among upregulated transcripts after treatment with ONC206 (0.5 µM) (**C**) or ONC212 (0.1 µM) (**D**) in DU145 and PC3 cells. Each polygon represents a Reactome pathway (grouped into super-pathways); color intensity encodes significance (−log10 FDR), and tile size reflects the number of mapped genes. Full statistics are provided in Supplementary Tables S4 and S5.

Reactome analysis of the top downregulated transcripts showed significant enrichment of terms related to DNA replication and cell-cycle programs (including G1/S transition) in DU145 and PC3 cells (DU145: *p* = 6.33 × 10^−15^ vs. ONC206, *p* = 1.44 × 10^−15^ vs. ONC212; PC3: *p* = 1.24 × 10^−10^ vs. ONC206, *p* = 2.92 × 10^−9^ vs. ONC212) (Figure 6A,B, Supplementary Table S4). Pathway analysis results highlighted coordinated downregulation of transcripts across replication machinery (PCNA, MCM helicases) and replication kinases (CDC7), central to origin licensing and S-phase entry (Supplementary Table S4). Interestingly, in PC3 cells, ONC206 and ONC212 showed enrichment of programs related to the activation/regulation of gene expression by SREBF (SREBP) and NR1H2/NR1H3, among downregulated gene sets, consistent with reduced expression of lipogenic and cholesterol-biosynthetic programs in this cell line (ONC206: *p* = 4.68 × 10^−7^ for activation of gene expression by SREBF (SREBP), *p* = 4.80 × 10^−6^ for regulation of cholesterol biosynthesis by SREBP (SREBF), *p* = 4.16 × 10^−5^ for NR1H2 and NR1H3 regulate gene expression linked to lipogenesis; ONC212: *p* = 4.4 × 10^−7^ for activation of gene expression by SREBF (SREBP), *p* = 4.52 × 10^−6^ for regulation of cholesterol biosynthesis by SREBP (SREBF), *p* = 4.04 × 10^−5^ for NR1H2 and NR1H3 regulate gene expression linked to lipogenesis) (Supplementary Figure S9 and Table S4). Analysis of the top upregulated transcripts revealed a shared stress-adaptive program across DU145 and PC3 cells with recurrent enrichment of the ISR (Response of EIF2AK1/HRI to heme deficiency) (DU145: *p* = 1.41 × 10^−9^ vs. ONC206, *p* = 1.62 × 10^−7^ vs. ONC212; PC3: *p* = 1.89 × 10^−10^ vs. ONC206, *p* = 1.24 × 10^−8^ vs. ONC212), UPR (DU145: *p* = 2.59 × 10^−5^ vs. ONC206, *p* = 7.71 × 10^−5^ vs. ONC212; PC3: *p* = 9.28 × 10^−6^ vs. ONC206, *p* = 1.81 × 10^−5^ vs. ONC212), and FOXO-mediated transcription of cell-cycle genes (DU145: *p* = 1.88 × 10^−5^ vs. ONC206, *p* = 5.17 × 10^−4^ vs. ONC212; PC3: *p* = 2.16 × 10^−7^ vs. ONC206, *p* = 7.57 × 10^−7^ vs. ONC212) (Figure 6C,D, Supplementary Table S5). These enrichments were accompanied by increased expression of canonical ISR/UPR and FOXO targets (*DDIT3*/*CHOP*, *ATF3*, *PPP1R15A*, *ASNS* and *CDKN1A*/*p21*, *GADD45A*, *BTG1*, *KLF4*) (Supplementary Table S5). Additionally, in DU145 cells, both compounds also prominently enriched p53-responsive transcription and senescence-associated modules (senescence-associated secretory phenotype and DNA damage/telomere-stress–induced senescence), indicating a shared stress- and growth-restraint program in this cell line (Supplementary Table S5). In PC3 cells, ONC206 showed stronger enrichment of PERK (EIF2AK3)–regulated gene expression within the UPR (*p* = 7.73 × 10^−6^), whereas ONC212 was associated with enrichment of mitochondrial RNA-metabolism pathways (tRNA and rRNA processing with *p* = 4.29 × 10^−5^ and 5.96 × 10^−5^, respectively) (Supplementary Figure S10 and Table S5).

Together, pathway enrichment analyses suggested transcriptional programs involving reduced expression of cell-cycle/DNA replication genes and enrichment of ISR/UPR and FOXO-linked stress-response signatures across AIPC cells.

### 2.5. ONC206 or ONC212 Induced DNA Fragmentation, Cell Cycle Perturbation, and Modulation of Mitochondrial Membrane Potential and Mass in Human AIPC Cells

**Figure 7:**
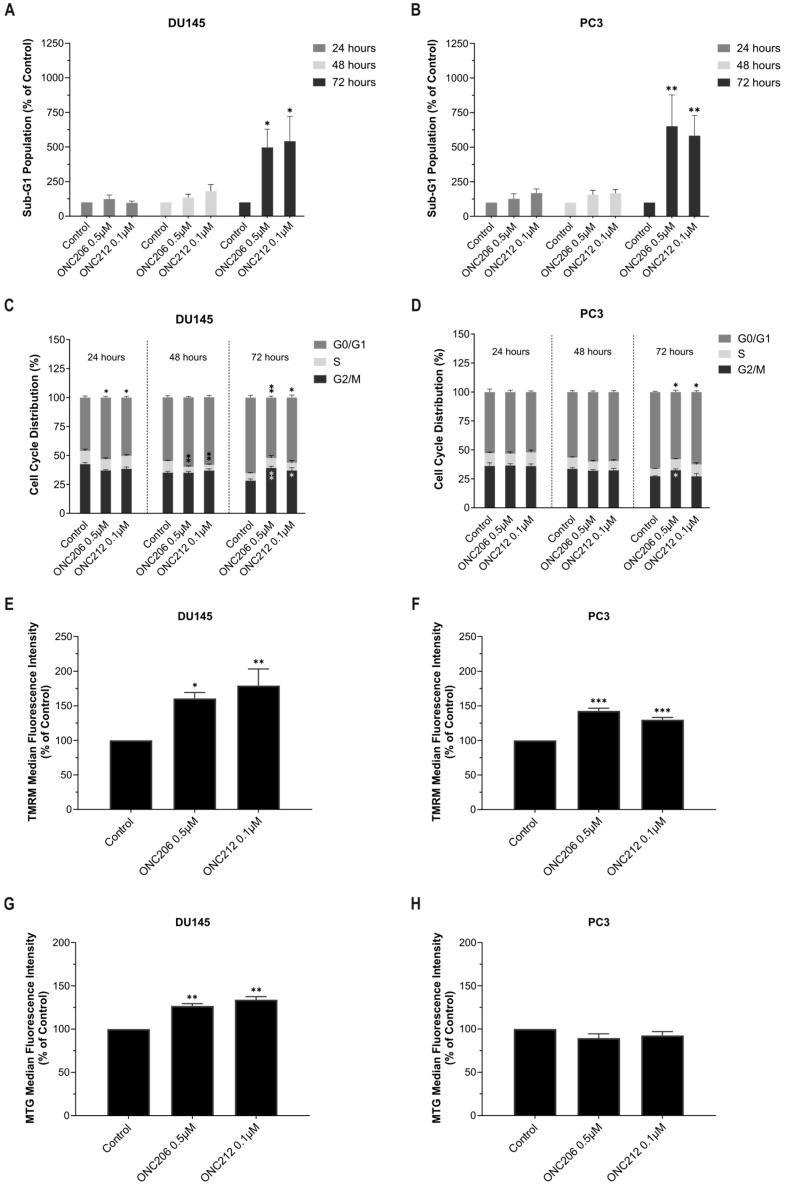
ONC206 or ONC212 induced cell-cycle redistribution, sub-G1 accumulation, and modulation of mitochondrial membrane potential and mass in androgen-independent prostate cancer cells. (**A**–**D**) DU145 and PC3 cells were treated with ONC206 (0.5 µM) or ONC212 (0.1 µM) for 24, 48, or 72 h. Cell cycle distribution was analyzed by propidium iodide (PI) staining and flow cytometry. (**A**,**B**) Bars show quantification of the sub-G1 fraction, normalized to the vehicle control at each time point. (**C**,**D**) Bars show the distribution of cells across G0/G1, S, and G2/M phases. Data represents the mean ± SEM of ≥ 4 independent experiments. (**E**–**H**) DU145 and PC3 cells were treated with ONC206 (0.5 µM) or ONC212 (0.1 µM) for 48 h. (**E**,**F**) Mitochondrial membrane potential was assessed by Tetramethylrhodamine, Methyl Ester, Perchlorate (TMRM) staining and flow cytometry. (**G**,**H**) Mitochondrial mass was evaluated using MitoTracker Green (MTG). Median fluorescence intensity (MFI) was normalized to vehicle control. Data Represents the mean ± SEM of ≥3 independent experiments. Statistical significance was determined by one- or two-way ANOVA with appropriate multiple-comparisons tests; * *p* < 0.05, ** *p* < 0.01, *** *p* < 0.001.

Based on the RNA-seq findings, cell-cycle distribution was further evaluated upon treatment with the corresponding GI_50_ concentrations of the imipridones using a propidium iodide (PI) assay. Treatment of DU145 and PC3 cells with each imipridone resulted in a time-dependent increase in the sub-G1 fraction, reaching at least a five-fold elevation at 72 h compared with the control (Figure 7A,B, Supplementary Figure S11A,B). Accordingly, significant DNA fragmentation—reflected by sub-G1 accumulation—was observed in both cell lines after 72 h of treatment with ONC201 (*p* < 0.0001 in DU145; *p* = 0.0003 in PC3), ONC206 (*p* = 0.0326 in DU145; *p* = 0.0017 in PC3), or ONC212 (*p* = 0.0326 in DU145; *p* = 0.0030 in PC3) (Figure 7A,B, Supplementary Figure S11A,B).

Moreover, treatment of DU145 cells with imipridones significantly increased the proportion of cells in the G0/G1 phase at 24 h (*p* = 0.0168 for ONC201 and ONC206; *p* = 0.0469 for ONC212) (Figure 7C, Supplementary Figure S11C). This effect persisted at 48 h and was accompanied by a significant reduction in the S-phase fraction (*p* = 0.0068 for ONC201; *p* = 0.0098 for ONC206 and ONC212) (Figure 7C, Supplementary Figure S11C). At 72 h, treatment with all three imipridones resulted in a significant accumulation of cells in the G2/M phase (*p* = 0.0071 for ONC201 and ONC206; *p* = 0.0273 for ONC212), together with a significant decrease in the G0/G1 fraction (*p* = 0.0357 for ONC201 and ONC206; *p* = 0.0055 for ONC212) (Figure 7C, Supplementary Figure S11C). In PC3 cells, imipridone treatment induced a distinct pattern of cell-cycle redistribution. At 48 h, ONC201 significantly reduced the S-phase fraction (*p* = 0.0311), without major alterations in G0/G1 or G2/M proportions (Supplementary Figure S11D). By 72 h, treatment with ONC201 or ONC206 resulted in a significant increase in the G2/M fraction compared with control (*p* = 0.0211 for ONC201; *p* = 0.0313 for ONC206) (Figure 7D, Supplementary Figure S11D). In parallel, all three imipridones significantly decreased the proportion of cells in the G0/G1 phase (*p* = 0.0232 for ONC201 and ONC206; *p* = 0.0343 for ONC212) (Figure 7D, Supplementary Figure S11D).

Together, these findings indicate that imipridones induce time-dependent cell cycle perturbations in AIPC cells, accompanied by significant sub-G1 accumulation. Upon treatment, DU145 cells display an early G0/G1 arrest followed by redistribution toward the G2/M compartment, whereas PC3 cells exhibit a delayed shift toward G2/M. These results complement the RNA-seq data and highlight cell line–specific cell cycle checkpoint responses to imipridone treatment.

Additionally, as RNA-seq analysis indicated activation of stress-response pathways with potential implications for mitochondrial metabolism, the effects of the three imipridones on mitochondrial membrane potential and mitochondrial mass were evaluated using Tetramethylrhodamine, Methyl Ester, Perchlorate (TMRM) and MitoTracker Green (MTG) stains, respectively. Treatment with ONC201 (5 µM), ONC206 (0.5 µM), or ONC212 (0.1 µM) for 48 h significantly increased the median fluorescence intensity (MFI) of TMRM to more than 160% and 130% in DU145 and PC3 cells, respectively (ONC201: *p* = 0.0179 in DU145; *p* < 0.0001 in PC3; ONC206: *p* = 0.0270 in DU145; *p* < 0.0001 in PC3; ONC212: *p* = 0.0066 in DU145; *p* = 0007 in PC3) (Figure 7E,F, Supplementary Figure S11E,F and S12A,B). In DU145 cells, the increase in TMRM signal was accompanied by a significant elevation in MTG fluorescence following 48 h treatment (*p* = 0017 for ONC201; *p* = 0073 for ONC206; *p* = 0037 for ONC212) (Figure 7G, Supplementary Figures S11G and S12A,B). In contrast, MTG MFI in PC3 cells remained almost unchanged after imipridone treatments (Figure 7H, Supplementary Figures S11H and S12A,B).

Interestingly, normalization of TMRM fluorescence to MTG intensity at the group level revealed an increase in mitochondrial membrane potential relative to mitochondrial mass in both AIPC cell lines (Supplementary Figure S12C,D). This effect was more pronounced in PC3 cells (TMRM/MTG: 1.40–1.61-fold), where mitochondrial mass remained largely unchanged across treatments (Supplementary Figure S12D), compared with DU145 cells (TMRM/MTG: 1.23–1.34-fold), in which the increase in membrane potential occurred in parallel with increased mitochondrial content (Supplementary Figure S12C).

## 3. Discussion

Across DU145 and PC3 AIPC models, next-generation imipridones, particularly ONC212, demonstrated greater potency, suppressing proliferation and viability, reducing migration, and abolishing PCa spheroid formation at nanomolar concentrations. These effects were accompanied by sub-G1 accumulation consistent with DNA fragmentation and coordinated cell cycle redistribution. Transcriptomic profiling revealed a convergent repression of DNA replication and cell cycle transition programs alongside activation of ISR/UPR and FOXO signaling pathways. Collectively, these findings position next-generation imipridones, especially ONC212, as promising candidates for biomarker-informed strategies targeting stress-response networks in PCa.

We first demonstrated that ONC206 and ONC212 inhibited the proliferation and viability of AIPC cells (DU145, PC3, and 22Rv1). Both compounds outperformed the first-in-class ONC201, with significant effects at nanomolar doses. However, ONC212 was consistently the most potent across the MTT, SRB, and trypan blue exclusion assays. Estimated by the SRB assay at 48 h in DU145 and PC3 cells, the mean GI_50_ for ONC212 was ~0.1 µM, approximately 5-fold lower than ONC206 (~0.5 µM) and 50-fold lower than ONC201 (~5 µM). This potency hierarchy aligns with the first ONC212 report, which prioritized the derivative for in vivo studies based on nanomolar, broad-spectrum activity and favorable tolerability [12]. Subsequent studies in additional models, including glioma and biliary tract cancer, likewise observed stronger antitumor activity of ONC212 relative to ONC201 or ONC206, consistent with our findings [31,32].

Transcriptomic profiling revealed coordinated repression of cell-cycle transition and DNA-replication programs with additional attenuation of DNA repair–related pathways, particularly in DU145 cells, together with activation of stress-response pathways, providing a molecular framework for the observed antiproliferative effects of imipridone derivatives. Functional validation using PI-based cell cycle analysis confirmed time- and context-dependent redistribution across phases. These findings indicate cell line–specific differences in the timing of checkpoint engagement following imipridone exposure. Our data are consistent with prior reports describing context-dependent cell-cycle arrest patterns upon treatment with imipridone derivatives. In ovarian and endometrial cancer models, ONC206 induced G0/G1 arrest accompanied by decreased S- and G2/M-phase fractions [24,33]. In acute myeloid leukemia models, ONC212 produced a pronounced G0/G1 arrest, whereas in solid tumor models such as pancreatic cancer, ONC212 induced context-dependent redistribution, including G2/M accumulation [29,34,35]. Collectively, these findings support the transcriptomic analysis and indicate that imipridones are associated with checkpoint perturbation and apoptosis-associated DNA fragmentation in a cancer model-dependent manner.

Notably, ONC212 also induced a transcriptomic signature consistent with altered mitochondrial RNA metabolism in AIPC cells, most evident in PC3, providing an additional mechanism by which the proliferative capacity may be curtailed at nanomolar doses. In the same datasets, we also observed enrichment of Hallmark hypoxia and heme-metabolism signatures, together with pathways linked to heme-deficiency–HRI signaling, a pattern frequently reported in settings of respiratory chain dysfunction/OXPHOS impairment and thus compatible with mitochondria-derived metabolic stress [36,37,38,39,40]. Further analyses of mitochondrial membrane potential and mitochondrial content suggested a hyperpolarization-like increase in mitochondrial membrane potential-associated signal, most prominently in PC3 cells, together with an increase in mitochondrial mass in DU145 cells following imipridone treatments. We also noted a phenol red shift toward yellow in imipridone-treated cultures, indicating extracellular acidification, which may accompany mitochondrial stress and altered cellular bioenergetics (Supplementary Figure S13). Most reports on other cancer models, including hepatocellular carcinoma, diffuse midline glioma, and serous endometrial cancer, have demonstrated a decrease in mitochondrial membrane potential, particularly with ONC206 treatment [22,41,42]. In line with our findings, a recent preprint by Tzaridis et al. reported that ONC201 and ONC206 induced a mitochondrial hyperpolarization paralleled by a decrease in ETC complexes and Survivin in medulloblastoma (MB) cells. The authors also reported a relative increase in cytochrome c release in ONC206-treated MB cells relative to ONC201, indicative of early apoptotic signaling [43]. ONC212’s effects on mitochondrial membrane potential appear context-dependent. In glioblastoma, ONC212 treatment suppressed mtDNA levels and decreased mitochondrial membrane potential [31], whereas in cervical and lung cancer cell lines, ONC212 induced apoptosis with no significant loss of mitochondrial membrane potential [29]. Cancer cells have been shown to maintain a hyperpolarized mitochondrial membrane potential compared to non-transformed cells as part of their metabolic rewiring, a feature that has been exploited for preferential accumulation of delocalized lipophilic cations and other mitochondria-targeted agents in tumor cells, thereby increasing cancer selectivity while comparatively sparing normal cells [44]. In several drug classes, an early transient increase in mitochondrial membrane potential has been observed under therapeutic stress, preceding subsequent mitochondrial dysfunction and collapse during execution of cell-death programs. In this context, the mitochondrial membrane potential increase observed here may reflect an early, context-specific state of mitochondrial distress that promotes ISR engagement and contributes to selective imipridone cytotoxicity. Notably, inner-membrane hyperpolarization has been proposed as one potential trigger of ISR signaling, including in settings of ATP synthase inhibition, although the underlying mechanism remains incompletely defined [14,45]. Taken together, these findings suggest that imipridone effects on mitochondrial membrane potential may be strongly dependent on the cancer model and warrant kinetic and functional follow-up.

In parallel, ONC212 was the only compound that sustained a significant reduction in migration at 48 h in both DU145 and PC3 cells, whereas the effects of ONC201 or ONC206 were no longer evident once non-treated control wounds closed. Reports on ONC212’s antimigratory activity have been context-dependent. Reduced migration has been described in malignancies, including chronic lymphocytic leukemia and uveal melanoma, but in HCT116 colorectal models, ONC212 did not inhibit migration, while ONC201 or ONC206 did [12,46,47]. These differences may have emerged from the lineage and genetic context of the cells used, such as p53/EMT status, as well as assay design and exposure. In our study, mitomycin C pre-treatment minimized proliferation, supporting a migration-specific effect of ONC212 at 0.1 µM. Follow-up mechanistic studies, including live-cell tracking, transwell assays, and focal-adhesion/cytoskeletal readouts, may clarify whether ONC212 primarily impacts motility, adhesion dynamics, or both.

Given the central role of PCSCs in intratumoral heterogeneity, therapy resistance, and relapse, we evaluated whether imipridone derivatives target PCSC subpopulations. In a 3D Matrigel™-based spheroid assay, which is a functional readout of stem/progenitor-like traits, all three compounds significantly reduced the spheroid-forming efficiency of DU145 and PC3 cells, with ONC212 showing the greatest potency. Notably, ONC212 completely abolished PCa spheroid formation at nanomolar concentrations, approximately 10 to 50-fold lower than those required for ONC206 or ONC201, highlighting enhanced efficacy against self-renewing, stem-like populations. Previous studies, predominantly from our group, have shown that ONC206 reduces sphere-forming potential in neuroblastoma, colorectal, and ovarian cancer models [23,26,48]. To our knowledge, this is the first demonstration of ONC206 or ONC212 activity against PCSCs, and the first report evaluating ONC212 in a 3D CSC context. Concordantly, RNA-seq revealed coordinated repression of stemness-associated transcripts across models, including *SOX2*, *MYB*, *PROM2*, and *IGFBP5*—together with induction of the growth-restraining factor *KLF4*, a transcriptomic pattern consistent with impaired self-renewal. Mechanistically, CSCs, including PCSCs, often exhibit a dependence on mitochondrial OXPHOS for survival and self-renewal; accordingly, perturbation of OXPHOS can selectively deplete CSC subpopulations. Given that imipridones disrupt mitochondrial metabolism and induce a hyperpolarized-like mitochondrial membrane potential signature in our AIPC models, these mitochondrial perturbations may contribute to the observed suppression in PCa spheroid formation [49,50,51].

Beyond the shared transcriptional backbone, particularly the stress-response signature, Imipridones’ downstream programs diverged in a cell line–dependent manner, underscoring context-dependent tumor heterogeneity. In DU145 cells, ONC206 and ONC212 prominently enriched p53/CDKN1A-responsive and senescence-associated transcriptional modules, including SASP and DNA damage/telomere-stress–induced senescence, which is suggestive of a predominantly cytostatic/senescence-like state, potentially co-existing with a cytotoxic fraction as indicated by the sub-G1 population. This interpretation aligns with reports that ONC201 and other ClpP agonists can elicit durable senescence-like growth arrest rather than purely acute cytotoxicity in several tumor models [28,40,52,53]. In contrast, PC3 cells showed marked downregulation of lipid- and cholesterol-regulatory programs in response to ONC206 and ONC212, including Reactome pathways related to SREBF-dependent transcription, cholesterol biosynthesis regulation by SREBP, and NR1H2/NR1H3 (LXR)-driven lipogenesis. This is notable given that SREBP1/2 were shown to be frequently upregulated in PCa and have been implicated in AR signaling, de novo lipogenesis, stem-like traits, metastasis, and resistance to therapies such as docetaxel. Additionally, pharmacological SREBP inhibition was shown to reduce prostate tumor growth and restore treatment sensitivity in preclinical models [54,55,56,57,58,59,60]. Alterations in SREBP–LXR signaling may occur downstream of mitochondrial dysfunction [61,62], and/or contribute upstream to the regulation of mitochondrial metabolism and cellular metabolic stress, and therefore warrant further investigation [63,64]. To our knowledge, suppression of SREBP–LXR–driven lipogenic and cholesterol-biosynthetic programs by imipridones has not been widely reported and may be particularly advantageous in lipogenesis-addicted PCa, which should be explored in future studies.

It is worth noting that DU145 cells harbor TP53 missense mutations, whereas PC3 cells are TP53-null [65,66,67]. Because neither cell line retains canonical wild-type (WT) p53 function, enrichment of “p53 pathway” gene sets in our RNA-seq is more appropriately interpreted as activation of stress-response modules, including ISR/UPR programs and p53-family/p73-associated transcriptional outputs, rather than classical WT p53 transactivation. This interpretation aligns with prior work showing that imipridones elicit an ATF4/CHOP-driven ISR and tumor cell death in a p53-independent manner across solid and hematologic models [24,35,49,68]. Accordingly, the transcriptional responses to ONC206 and ONC212 observed here are consistent with a WT p53–nonfunctional background, supporting a model in which imipridone efficacy is mediated primarily through ISR engagement rather than canonical WT p53 activation.

We acknowledge several limitations. First, we profiled only two human AIPC models (DU145 and PC3), both with defective TP53 and low-to-absent AR activity; thus, generalizability to AR-driven adenocarcinoma or neuroendocrine disease remains uncertain. Second, although imipridones have shown tumor-selective activity and favorable tolerability profiles in prior preclinical studies and early-phase clinical reports [12,33,35,69,70,71,72], since we did not include non-malignant prostate epithelium controls, tumor selectivity and cytostatic versus cytotoxic effects in normal cells were not defined in this study and should be addressed in future work. Third, mechanistic conclusions are inferred from transcriptomics without causal perturbation, such as ClpP loss-of-function, DRD2/GPR132 modulation, or orthogonal metabolic and DNA-repair assays. Fourth, our analysis reflects limited dose and time windows (mainly 48 h post-treatment; 0.5 µM ONC206 and 0.1 µM ONC212), warranting broader kinetic and dose–response follow-up. Finally, since our findings are derived from in vitro models, in vivo validation remains essential to confirm pharmacodynamic target engagement, therapeutic efficacy, and safety in the context of the tumor microenvironment.

Collectively, our data indicates that ONC206 and ONC212 share an ISR-centered mode of action in DU145 and PC3 AIPC cells but resolve this stress through distinct, context-dependent programs. In DU145 cells, imipridones preferentially enrich p53/CDKN1A-linked and senescence-associated modules, consistent with a predominantly cytostatic growth-restraint state with senescence-like features. In PC3 cells, by contrast, both compounds suppress SREBP–LXR–driven lipogenic and cholesterol-biosynthetic programs, and ONC212 additionally induces a mitochondrial RNA/translation signature, suggesting a distinct mitochondrial RNA-metabolism vulnerability in this line. Together, these responses underscore PCa heterogeneity and illustrate how convergent ISR/UPR engagement, potentially driven by distinct upstream perturbations, can yield divergent transcriptional and phenotypic outcomes even across advanced AIPC models, motivating targeted functional studies and in vivo validation.

## 4. Materials and Methods

### 4.1. Cell Culture Conditions

DU145, PC3, and 22Rv1 human PCa cell lines were obtained from the American Type Culture Collection (ATCC, Manassas, VA, USA). Cells were cultured in RPMI 1640 medium (Sigma-Aldrich, St. Louis, MO, USA) supplemented with 10% fetal bovine serum (FBS; Sigma-Aldrich), 1% penicillin-streptomycin (Sigma-Aldrich), and Plasmocin^®^ prophylactic (InvivoGen, San Diego, CA, USA). Cultures were maintained at 37 °C in a humidified atmosphere containing 5% CO_2_ and were routinely confirmed to be mycoplasma-free.

### 4.2. Drug Preparation

ONC201, ONC206, and ONC212 were obtained from Chimerix (Chimerix, Durham, NC, USA). Compounds were reconstituted in dimethyl sulfoxide (DMSO; Sigma-Aldrich) according to the manufacturer’s instructions. Stock solutions (20 mM) were stored at −20 °C. Vehicle controls were DMSO-matched to the corresponding treatment conditions. The final DMSO concentration did not exceed 0.05% (*v*/*v*), corresponding to the highest drug concentration tested (10 µM).

### 4.3. MTT Assay

The anti-proliferative effect of imipridones was assessed using the MTT assay [3-(4,5-dimethylthiazol-2-yl)-2,5-diphenyltetrazolium bromide; Sigma-Aldrich]. Briefly, 5000 PCa cells were seeded in triplicate into 96-well plates and incubated overnight. Cells were then treated with increasing concentrations of imipridones for 24, 48, or 72 h. At each time point, the medium was replaced with 5 mg/mL MTT reagent (Sigma-Aldrich) prepared in culture medium. Following a 3 h incubation at 37 °C, the MTT solution was discarded and replaced with absolute isopropanol (Sigma-Aldrich) to solubilize the formazan crystals. Absorbance of the samples was measured at 595 nm using a Tristar Multimode Reader (Berthold Technologies GmbH & Co. KG, Bad Wildbad, Germany), with background correction by subtraction of the blank wells (no cells) reading. Cell proliferation was expressed as a percentage relative to the DMSO-treated control group.

### 4.4. SRB Assay

The SRB assay was used to evaluate the cytotoxic effects of imipridones on PCa cells as described [73]. Briefly, 5000 PCa cells were seeded in triplicate into 96-well plates and treated with increasing concentrations of imipridones for 24, 48, or 72 h. At each time point, cold 50% trichloroacetic acid (TCA; Sigma-Aldrich) solution was gently added to each well, and plates were incubated at 4 °C for 1 h. Wells were then washed four times with double-distilled water (ddH_2_O) and allowed to air dry at room temperature (RT). A 0.04% SRB solution (Sigma-Aldrich) was added to each well and incubated for 1 h at RT. Plates were subsequently rinsed four times with 1% acetic acid (Sigma-Aldrich) and allowed to air-dry again. A 10 mM Tris base (Bio-Rad, Hercules, CA, USA) solution was added to solubilize the protein-bound dye. Absorbance of the samples was measured at 510 nm using a Tristar Multimode Reader, with background correction by subtraction of the blank wells (no cells) reading. Cell growth was expressed as a percentage relative to the DMSO-treated control group. GI_50_ values were calculated by nonlinear regression of SRB dose–response curves (log[inhibitor] vs. normalized response) using GraphPad Prism version 10.4.2 (GraphPad Software, San Diego, CA, USA). When GI_50_ was not reached within the tested range, values were reported as greater than the maximum tested concentration.

### 4.5. Trypan Blue Exclusion Assay

The effects of imipridones on PCa cell viability were assessed using the trypan blue exclusion assay. 30,000 PCa cells were seeded in duplicate in 24-well plates and incubated overnight. Then, cells were treated with increasing concentrations of imipridones. After 48 h, the attached cells were harvested and mixed with trypan blue dye (Sigma-Aldrich) at a 1:1 ratio. Viable cells were counted using a hemocytometer under the Axiovert microscope (Carl Zeiss Microscopy, Oberkochen, Germany). Cell viability was expressed as a percentage relative to the DMSO-treated control group.

### 4.6. Wound Healing (Scratch) Assay

The scratch assay was used to assess the effects of imipridones on PCa cell migration. Near-confluent PCa cells, seeded in 24-well plates, were treated with 10 µg/mL mitomycin C (Sigma-Aldrich) for 10 min to inhibit proliferation. A uniform scratch was created using a sterile 200 µL micropipette tip (Corning Inc., Corning, NY, USA), followed by the addition of treatment conditions. Images of the wound area were captured at 0, 24, and 48 h post-treatment using an Axiovert microscope. Wound areas were quantified at each time point using ImageJ 1.54g software (National Institutes of Health, Bethesda, MD, USA). The percentage of wound area was reported according to [74] as below:

Migration rate (%) or Wound Closure (%) = [(A_t=0h_ − A_t=Δh_)/A_t=0h_] × 100%

A_t=0h_ = wound area at 0 h.

A_t=Δh_ = wound area h hours after the scratch is performed.

### 4.7. Sphere-Formation Assay

The 3D sphere-formation assay was used to assess the ability of imipridones to target PCa stem/progenitor-like cells in DU145 and PC3 cell lines, as previously described [75]. Briefly, 1000 PCa cells per well were suspended in FBS-free medium and mixed 1:1 with cold growth factor-reduced Matrigel™. The cell/Matrigel™ mixture was seeded around the rim of each well in 96-well plates, which was then incubated at 37 °C for at least 30 min to allow the Matrigel™ to solidify. Warm RPMI 1640 medium supplemented with 5% FBS, with or without imipridone treatments, was gently added to each well. Medium was replenished every other day. After 7–8 days, formed spheres were counted and imaged using an Axiovert microscope. The percentage of SFU was calculated by dividing the number of spheres formed per condition by the number of cells originally seeded and expressed as a percentage relative to the DMSO-treated control group. The average sphere area was determined using ZEN 3.11 software (Carl Zeiss Microscopy, Oberkochen, Germany). Specifically, after acquisition of bright-field images, representative images containing at least 30 spheres per condition were analyzed using the Ellipse measurement tool to fit each sphere and extract the 2D projected area (µm^2^); values were then averaged per condition. Average sphere area was expressed as a percentage relative to the DMSO-treated control group.

### 4.8. RNA Extraction and Gel Electrophoresis

Total RNA was extracted from PCa cells, treated or untreated with imipridones, using the RNeasy Plus Mini Kit (QIAGEN, Hilden, Germany) according to the manufacturer’s instructions. RNA concentration and purity were assessed using a DeNovix DS-11 spectrophotometer (DeNovix Inc., Wilmington, DE, USA), and RNA integrity was evaluated by agarose gel electrophoresis. For RNA electrophoresis, a 1% agarose gel was prepared in 1× Tris-acetate-EDTA (TAE) buffer containing ethidium bromide (EtBr). Once the gel had solidified in the casting tray, TAE buffer was added to the electrophoresis chamber, and 1 µg of each RNA sample was loaded. Electrophoresis was carried out until the samples migrated approximately two-thirds of the gel length. RNA integrity was confirmed by the presence of sharp 28S and 18S ribosomal RNA bands.

### 4.9. Whole Transcriptome Sequencing Analysis

RNA-seq was performed at Macrogen Inc. (Seoul, Republic of Korea). RNA-seq data in FASTQ format were first assessed for quality using FastQC, and potential contamination was evaluated with FastQ Screen [76,77]. High-quality reads were then aligned to the *Homo sapiens* reference genome (hg38) using the splice-aware aligner HISAT2 v2.2.1 [78]. Resulting SAM files were converted to sorted BAM files by genomic coordinates using SAMtools v1.20 [79]. Gene-level read quantification was performed with featureCounts v2.0.6 using GENCODE v48 annotation (GTF file) and the following parameters: --countReadPairs -s 2 -p -B -g gene_symbol -t exon [80]. The resulting count matrix was imported into R (Bioconductor v3.19) and differential expression analysis was carried out using the DESeq2 v1.44.0 package from Bioconductor [81]. Genes with an absolute log_2_ fold change ≥ 1 and adjusted *p*-value < 0.05 were considered significant and were used for further analysis. Pathways enrichment analysis of the dysregulated genes was conducted using enrichR online web tool (Ma’ayan Laboratory, Icahn School of Medicine at Mount Sinai, New York, NY, USA) and Reactome gene sets from the Reactome Pathway Database (Reactome Team; hosted at EMBL-EBI, Hinxton, Cambridgeshire, UK [82]. ComplexHeatmap v2.20.0 [83], ggplot2 v3.5.1 [84], and eulerr v7.0.2 [85] R packages were used to generate the heatmap, PCA plot, volcano plot, and the Venn diagrams.

### 4.10. Reverse Transcription (RT) Quantitative Real-Time PCR (qPCR)

Selected DEGs, identified from RNA-seq data, were validated by qRT-PCR. RT of 1 µg of total RNA (extracted as described above) into complementary DNA (cDNA) was performed using the QuantiTect Reverse Transcription Kit (Qiagen), according to the manufacturer’s instructions. Amplification was performed using SYBR Green PCR Master Mix and the CFX384 Touch™ Real-Time PCR Detection System (Bio-Rad). All reactions were run in duplicate using gene-specific primers listed in Table 1. The thermal cycling conditions were set to an initial denaturation at 95 °C for 5 min, followed by 40 cycles of 95 °C for 10 s, 60 °C for 30 s, and 72 °C for 30 s. Relative expression was calculated using the comparative Ct method (ΔΔCt). Gene expression levels were normalized separately to the housekeeping genes *ACTB* and *GAPDH*, and the final reported value represents the arithmetic mean of the two resulting fold-change values.

### 4.11. Cell Cycle and Sub-G1 Analysis by PI Staining

PI staining was used to assess the effects of imipridones on cell cycle distribution. PCa cells were seeded in 6-well plates and treated with the indicated concentrations for 24, 48, or 72 h. At each time point, both floating and adherent cells were collected and fixed in ice-cold 70% ethanol (Sigma-Aldrich). On the day of analysis, fixed cells were incubated with DNase-free RNase A (200 µg/mL; Qiagen), followed by staining with PI solution (70 µg/mL). Fluorescence was measured using a Guava EasyCyte™ 8 Flow Cytometer (Millipore, Billerica, MA, USA). Sub-G1 accumulation and cell cycle distribution (G0/G1, S, and G2/M phases) were analyzed using GuavaSoft™ v2.7 (Millipore, Billerica, MA, USA) software. Sub-G1 fractions were expressed as a percentage relative to the DMSO-treated control group, whereas G0/G1, S, and G2/M phases were expressed as percentages of the total cell population.

### 4.12. Mitochondrial Membrane Potential Analysis by TMRM Staining

To assess the effects of imipridones on the mitochondrial membrane potential, TMRM staining was performed. PCa cells were seeded in 6-well plates and treated with the indicated concentrations for 48 h. Both floating and adherent cells were collected and incubated with TMRM potentiometric dye (100 nM; Invitrogen, Thermo Fisher Scientific, Waltham, MA, USA) for 30 min at 37 °C. Fluorescence was immediately measured using a Guava EasyCyte™ 8 Flow Cytometer. Carbonyl cyanide p-(trifluoromethoxy)phenylhydrazone (FCCP; 2 µM) was incubated for 10 min at 37 °C after TMRM initial reading as a positive control for mitochondrial depolarization. TMRM MFI was quantified using GuavaSoft™ 2.7 software. Mitochondrial membrane potential was expressed as a percentage relative to the DMSO-treated control group.

### 4.13. Mitochondrial Mass Analysis by MTG Staining

The effects of imipridones on mitochondrial mass were assessed using MTG staining. PCa cells were seeded in 6-well plates and treated with the indicated concentrations for 48 h. Both floating and adherent cells were collected and incubated with MTG (100 nM; Invitrogen) for 20 min at 37 °C. Fluorescence was measured using a Guava EasyCyte™ 8 Flow Cytometer. MTG MFI was quantified using GuavaSoft™ 2.7 software. Mitochondrial Mass was expressed as a percentage relative to the DMSO-treated control group.

### 4.14. Statistical Analysis

Statistical analyses were performed using GraphPad Prism version 10.4. Data is presented as mean ± standard error of the mean (SEM) from at least three independent experiments, unless otherwise stated. Comparisons between experimental groups were made using one- or two-way ANOVA, as appropriate. A *p*-value of <0.05 was considered statistically significant.

## 5. Conclusions

ONC212 demonstrated robust in vitro activity against key tumor-promoting processes in advanced, AIPC—suppressing proliferation and migration and eradicating PCa spheroids at nanomolar concentrations—and consistently outperformed ONC201 and ONC206 across 2D and 3D assays. To our knowledge, this is the first report of ONC212 activity in 3D sphere cultures and the first RNA-seq profiling of ONC206 and ONC212 in AIPC, defining a shared ISR/UPR-anchored, cell cycle–suppressive transcriptional backbone with drug-specific, non-redundant biases. Collectively, these findings position ONC212 as a lead imipridone candidate for therapy-refractory PCa and provide a practical framework—and public RNA-seq resource—for biomarker-guided combinations and in vivo validation, supporting progression toward translational studies.

## Figures and Tables

**Table 1:** Primer sequences used for reverse transcription quantitative real-time PCR (qRT-PCR). Forward and reverse primers are listed in the 5′-Oligo Seq-3′ direction with their corresponding target genes.

Gene Symbol	Forward Primer Sequence	Reverse Primer Sequence
*PPP1R15A*	TCCTCTGGCAATCCCCCATA	TGGTTTTCAGCCCCAGTGTT
*ERN1*	ACGCCCACTCTGTATGTTGG	CAAACTTGACGTCCGTGCTG
*CDKN2C*	TCACCACCGTGAACAAGGG	GCAGTCTCCTGGCAATCTCG
*RRM2*	TTGGTGGAGCGATTTAGCCA	GCCCAGTCTGCCTTCTTCTT
*ACTB*	CTCACCATGGATGATGATATCGC	AGGAATCCTTCTGACCCATGC
*GAPDH*	GTCAGTGGTGGACCTGACCT	TCGCTGTTGAAGTCAGAGGA

## Data Availability

All data generated or analyzed during this study are included in this published article and its supplementary information files. The RNA-seq datasets have been deposited in the Gene Expression Omnibus (GEO) under the accession number GSEXXXXXX and will be released upon publication.

## Supplementary Materials

The following supporting information can be downloaded at: https://www.mdpi.com/article/10.3390/ijms27104597/s1.

## Abbreviations

The following abbreviations are used in this manuscript:

PCa	Prostate cancer
PCSCs	Prostate cancer stem cells
AIPC	Androgen-independent prostate cancer
ISR	Integrated stress response
UPR	Unfolded protein response
mCRPC	Metastatic castration-resistant prostate cancer
AR	Androgen receptor
TRAIL	Tumor necrosis factor-related apoptosis-inducing ligand
GPCRs	G protein-coupled receptors
DRD2	Dopamine Receptor D2
ClpP	Caseinolytic protease P
ETC	Electron transport chain
OXPHOS	Oxidative phosphorylation
CNS	Central nervous system
2D	Two-dimensional
3D	Three-dimensional
MTT	Thiazolyl blue tetrazolium bromide
SRB	Sulforhodamine B
GI_50_	Growth inhibitory concentration 50%
SFU	Sphere-forming unit
RNA-seq	RNA-sequencing
DEGs	Differentially expressed genes
PCA	Principal component analysis
PI	Propidium iodide
TMRM	Tetramethylrhodamine, methyl ester, perchlorate
MTG	Mitotracker Green
MFI	Median fluorescence intensity
MB	Medulloblastoma
WT	Wild-type
ATCC	American type culture collection
FBS	Fetal bovine serum
DMSO	Dimethyl sulfoxide
TCA	Trichloroacetic acid
RT	Room temperature
TAE	Tris-acetate-EDTA
EtBr	Ethidium bromide
RT	Reverse transcription
qPCR	Quantitative real-time polymerase chain reaction
cDNA	Complementary DNA
FCCP	Carbonyl cyanide p-(trifluoromethoxy)phenylhydrazone
SEM	Standard error of the mean

## References

[1] Bray F., Laversanne M., Sung H., Ferlay J., Siegel R.L., Soerjomataram I., Jemal A. (2024). Global cancer statistics 2022: GLOBOCAN estimates of incidence and mortality worldwide for 36 cancers in 185 countries. CA Cancer J. Clin..

[2] Tolkach Y., Kristiansen G. (2018). The Heterogeneity of Prostate Cancer: A Practical Approach. Pathobiology.

[3] Haffner M.C., Zwart W., Roudier M.P., True L.D., Nelson W.G., Epstein J.I., De Marzo A.M., Nelson P.S., Yegnasubramanian S. (2021). Genomic and phenotypic heterogeneity in prostate cancer. Nat. Rev. Urol..

[4] Wolf I., Gratzke C., Wolf P. (2022). Prostate Cancer Stem Cells: Clinical Aspects and Targeted Therapies. Front. Oncol..

[5] Zhou K., Lu H., Zhang J., Shen Q., Liu P., Xu Q., Yang C., Mao L. (2024). Prostate cancer stem cells: An updated mini-review. J. Cancer.

[6] Karantanos T., Corn P.G., Thompson T.C. (2013). Prostate cancer progression after androgen deprivation therapy: Mechanisms of castrate resistance and novel therapeutic approaches. Oncogene.

[7] Paolo C., Giorgia Z. (2021). Metabolic reprogramming as an emerging mechanism of resistance to endocrine therapies in prostate cancer. Cancer Drug Resist..

[8] Imamura J., Ganguly S., Muskara A., Liao R.S., Nguyen J.K., Weight C., Wee C.E., Gupta S., Mian O.Y. (2023). Lineage plasticity and treatment resistance in prostate cancer: The intersection of genetics, epigenetics, and evolution. Front. Endocrinol..

[9] Fontana F., Anselmi M., Limonta P. (2023). Unraveling the Peculiar Features of Mitochondrial Metabolism and Dynamics in Prostate Cancer. Cancers.

[10] Papadaki S., Magklara A. (2022). Regulation of Metabolic Plasticity in Cancer Stem Cells and Implications in Cancer Therapy. Cancers.

[11] O’Malley J., Kumar R., Inigo J., Yadava N., Chandra D. (2020). Mitochondrial stress response and cancer. Trends Cancer.

[12] Wagner J., Kline C.L., Ralff M.D., Lev A., Lulla A., Zhou L., Olson G.L., Nallaganchu B.R., Benes C.H., Allen J.E. (2017). Preclinical evaluation of the imipridone family, analogs of clinical stage anti-cancer small molecule ONC201, reveals potent anti-cancer effects of ONC212. Cell Cycle.

[13] Prabhu V.V., Morrow S., Rahman Kawakibi A., Zhou L., Ralff M., Ray J., Jhaveri A., Ferrarini I., Lee Y., Parker C. (2020). ONC201 and imipridones: Anti-cancer compounds with clinical efficacy. Neoplasia.

[14] Bonner E.R., Waszak S.M., Grotzer M.A., Mueller S., Nazarian J. (2021). Mechanisms of imipridones in targeting mitochondrial metabolism in cancer cells. Neuro-Oncol..

[15] Ishizawa J., Zarabi S.F., Davis R.E., Halgas O., Nii T., Jitkova Y., Zhao R., St-Germain J., Heese L.E., Egan G. (2019). Mitochondrial ClpP-mediated proteolysis induces selective cancer cell lethality. Cancer Cell.

[16] Allen J.E., Kline C.L.B., Prabhu V.V., Wagner J., Ishizawa J., Madhukar N., Lev A., Baumeister M., Zhou L., Lulla A. (2016). Discovery and clinical introduction of first-in-class imipridone ONC201. Oncotarget.

[17] Szász Z., Takács A., Kalabay M., Bárány P., Czuczi T., Csámpai A., Lajkó E., Kőhidai L. (2025). Comparative study of the anti-tumour effects of the imipridone, ONC201 and its fluorinated analogues on pancreatic cancer cell line. Sci. Rep..

[18] Lev A., Lulla A.R., Ross B.C., Ralff M.D., Makhov P.B., Dicker D.T., El-Deiry W.S. (2018). ONC201 Targets AR and AR-V7 Signaling, Reduces PSA, and Synergizes with Everolimus in Prostate Cancer. Mol. Cancer Res..

[19] National Center for Biotechnology Information PubChem Compound Summary for CID 73777259, Dordaviprone. https://pubchem.ncbi.nlm.nih.gov/compound/Dordaviprone.

[20] National Center for Biotechnology Information PubChem Compound Summary for CID 135297777, ONC-206. https://pubchem.ncbi.nlm.nih.gov/compound/135297777.

[21] National Center for Biotechnology Information PubChem Compound Summary for CID 124085867, ONC-212. https://pubchem.ncbi.nlm.nih.gov/compound/124085867.

[22] Zhang Y., Huang Y., Yin Y., Fan Y., Sun W., Zhao X., Tucker K., Staley A., Paraghamian S., Hawkins G. (2020). ONC206, an Imipridone Derivative, Induces Cell Death Through Activation of the Integrated Stress Response in Serous Endometrial Cancer In Vitro. Front. Oncol..

[23] El-Soussi S., Hanna R., Semaan H., Khater A.-R., Abdallah J., Abou-Kheir W., Abou-Antoun T. (2021). A novel therapeutic mechanism of imipridones ONC201/ONC206 in MYCN-amplified neuroblastoma cells via differential expression of tumorigenic proteins. Front. Pediatr..

[24] Staley A., Tucker K., Yin Y., Zhang X., Fan Y., Zhang Y., Fang Z., Sun W., Suo H., Zhao X. (2021). Highly potent dopamine receptor D2 antagonist ONC206 demonstrates anti-tumorigenic activity in endometrial cancer. Am. J. Cancer Res..

[25] Przystal J.M., Cianciolo Cosentino C., Yadavilli S., Zhang J., Laternser S., Bonner E.R., Prasad R., Dawood A.A., Lobeto N., Chin Chong W. (2022). Imipridones affect tumor bioenergetics and promote cell lineage differentiation in diffuse midline gliomas. Neuro-Oncol..

[26] Monzer A., Ghamlouche F., Wakimian K., Ballout F., Al Bitar S., Yehya A., Kanso M., Saheb N., Tawil A., Doughan S. (2025). ONC206, an imipridone derivative, demonstrates anti-colorectal cancer activity against stem/progenitor cells in 3D cell cultures and in patient-derived organoids. Pharmacol. Rep..

[27] Ferrarini I., Louie A., Zhou L., El-Deiry W.S. (2021). ONC212 is a Novel Mitocan Acting Synergistically with Glycolysis Inhibition in Pancreatic Cancer. Mol. Cancer Ther..

[28] Wedam R., Greer Y.E., Wisniewski D.J., Weltz S., Kundu M., Voeller D., Lipkowitz S. (2023). Targeting Mitochondria with ClpP Agonists as a Novel Therapeutic Opportunity in Breast Cancer. Cancers.

[29] Basu V., Shabnam, Murghai Y., Ali M., Sahu S., Verma B.K., Seervi M. (2024). ONC212, alone or in synergistic conjunction with Navitoclax (ABT-263), promotes cancer cell apoptosis via unconventional mitochondrial-independent caspase-3 activation. Cell Commun. Signal..

[30] Chi A.S. (2017). Identification of more potent imipridones, a new class of anti-cancer agents. Cell Cycle.

[31] Ishida C.T., Zhang Y., Bianchetti E., Shu C., Nguyen T.T., Kleiner G., Sanchez-Quintero M.J., Quinzii C.M., Westhoff M.-A., Karpel-Massler G. (2018). Metabolic reprogramming by dual AKT/ERK inhibition through imipridones elicits unique vulnerabilities in glioblastoma. Clin. Cancer Res..

[32] Saxena A., Lannigan A.J., Sun G., Chan J., Trainor J., Zhou L., Prabhu V.V., El-Deiry W.S., Raufi A.G. (2025). Imipridones ONC201, ONC206, and ONC212 promote immune-mediated cell death and anti-tumor activity in biliary tract cancer models in vitro. Cancer Res..

[33] Tucker K., Yin Y., Staley S.A., Zhao Z., Fang Z., Fan Y., Zhang X., Suo H., Sun W., Prabhu V.V. (2022). ONC206 has anti-tumorigenic effects in human ovarian cancer cells and in a transgenic mouse model of high-grade serous ovarian cancer. Am. J. Cancer Res..

[34] Lev A., Lulla A.R., Wagner J., Ralff M.D., Kiehl J.B., Zhou Y., Benes C.H., Prabhu V.V., Oster W., Astsaturov I. (2017). Anti-pancreatic cancer activity of ONC212 involves the unfolded protein response (UPR) and is reduced by IGF1-R and GRP78/BIP. Oncotarget.

[35] Nii T., Prabhu V.V., Ruvolo V., Madhukar N., Zhao R., Mu H., Heese L., Nishida Y., Kojima K., Garnett M.J. (2019). Imipridone ONC212 activates orphan G protein-coupled receptor GPR132 and integrated stress response in acute myeloid leukemia. Leukemia.

[36] Evstafieva A., Garaeva A., Khutornenko A., Klepikova A., Logacheva M., Penin A., Novakovsky G., Kovaleva I., Chumakov P. (2014). A sustained deficiency of mitochondrial respiratory complex III induces an apoptotic cell death through the p53-mediated inhibition of pro-survival activities of the activating transcription factor 4. Cell Death Dis..

[37] Sasaki K., Uchiumi T., Toshima T., Yagi M., Do Y., Hirai H., Igami K., Gotoh K., Kang D. (2020). Mitochondrial translation inhibition triggers ATF4 activation, leading to integrated stress response but not to mitochondrial unfolded protein response. Biosci. Rep..

[38] Suragani R.N., Zachariah R.S., Velazquez J.G., Liu S., Sun C.W., Townes T.M., Chen J.J. (2012). Heme-regulated eIF2α kinase activated Atf4 signaling pathway in oxidative stress and erythropoiesis. Blood.

[39] Chen J.J. (2025). HRI protein kinase in cytoplasmic heme sensing and mitochondrial stress response: Relevance to hematological and mitochondrial diseases. J. Biol. Chem..

[40] Armenise D., Baldelli O.M., Liturri A., Cavallaro G., Fortuna C.G., Ferorelli S., Miciaccia M., Perrone M.G., Scilimati A. (2025). Mitochondrial Protease ClpP: Cancer Marker and Drug Target. Pharmaceuticals.

[41] Cao J., Cao F., Wang C., Jiao Z., You Y., Wang X., Zhao W. (2024). ONC206 targeting ClpP induces mitochondrial dysfunction and protective autophagy in hepatocellular carcinoma cells. Neoplasia.

[42] Purow B. (2022). ONC201 and ONC206: Metabolically ClipPing the wings of diffuse midline glioma. Neuro-Oncol..

[43] Tzaridis T., Liu J., Chien F.L., Malhotra A., Zhu D., Gershon I., Zhang H., Velazquez Vega J.E., Schniederjan M., Sposito T. (2025). ONC206 demonstrates potent anti-tumorigenic activity and is a potential novel therapeutic strategy for high-risk medulloblastoma. bioRxiv.

[44] Nguyen C., Pandey S. (2019). Exploiting Mitochondrial Vulnerabilities to Trigger Apoptosis Selectively in Cancer Cells. Cancers.

[45] Mick E., Titov D.V., Skinner O.S., Sharma R., Jourdain A.A., Mootha V.K. (2020). Distinct mitochondrial defects trigger the integrated stress response depending on the metabolic state of the cell. eLife.

[46] Rizwan N., Shen Y., Iwanowicz E., Mulligan S.P., Crassini K.R., Christopherson R., Best O.G. (2018). ONC-212 (I-39), a Novel Inhibitor of the UPR, Is Cytotoxic and Cytostatic Against CLL Cells Under in Vitro Conditions That Mimic the Tumor Microenvironment. Blood.

[47] Chattopadhyay C., Roszik J., Bhattacharya R., Alauddin M., Mahmud I., Yadugiri S., Ali M.M., Khan F.S., Prabhu V.V., Lorenzi P.L. (2024). Imipridones inhibit tumor growth and improve survival in an orthotopic liver metastasis mouse model of human uveal melanoma. Br. J. Cancer.

[48] Mikhael S., Fayyad R., Harfouch L.A., Prabhu V.V., Bahmad H.F., Abou-Kheir W., Daoud G. (2025). Investigating the Effects of ONC206 Alone and in Combination with Cisplatin on Ovarian Cancer Cell Models. Curr. Issues Mol. Biol..

[49] Sancho P., Barneda D., Heeschen C. (2016). Hallmarks of cancer stem cell metabolism. Br. J. Cancer.

[50] Ahmad F., Cherukuri M.K., Choyke P.L. (2021). Metabolic reprogramming in prostate cancer. Br. J. Cancer.

[51] Jones C.L., Inguva A., Jordan C.T. (2021). Targeting Energy Metabolism in Cancer Stem Cells: Progress and Challenges in Leukemia and Solid Tumors. Cell Stem Cell.

[52] Mishukov A., Odinokova I., Mndlyan E., Kobyakova M., Abdullaev S., Zhalimov V., Glukhova X., Galat V., Galat Y., Senotov A. (2022). ONC201-induced mitochondrial dysfunction, senescence-like phenotype, and sensitization of cultured BT474 human breast cancer cells to TRAIL. Int. J. Mol. Sci..

[53] Aponte-Collazo L.J., Fennell E.M., East M.P., Gilbert T.S., Graves P.R., Ashamalla H., Iwanowicz E.J., Greer Y.E., Lipkowitz S., Graves L.M. (2022). Small molecule ClpP agonists induce senescence and alter TRAIL-mediated apoptotic response of triple-negative breast cancer cells. bioRxiv.

[54] Li X., Wu J.B., Li Q., Shigemura K., Chung L.W., Huang W.C. (2016). SREBP-2 promotes stem cell-like properties and metastasis by transcriptional activation of c-Myc in prostate cancer. Oncotarget.

[55] Brandt M.P., Vakhrusheva O., Hackl H., Daher T., Tagscherer K., Roth W., Tsaur I., Handle F., Eigentler A., Culig Z. (2024). Inhibition of the Sterol Regulatory Element Binding Protein SREBF-1 Overcomes Docetaxel Resistance in Advanced Prostate Cancer. Am. J. Pathol..

[56] Huang W.-C., Li X., Liu J., Lin J., Chung L.W. (2012). Activation of androgen receptor, lipogenesis, and oxidative stress converged by SREBP-1 is responsible for regulating growth and progression of prostate cancer cells. Mol. Cancer Res..

[57] Parupathi P., Devarakonda L.S., Francois E., Amjed M., Kumar A. (2025). Reprogrammed Lipid Metabolism-Associated Therapeutic Vulnerabilities in Prostate Cancer. Int. J. Mol. Sci..

[58] Chen J., Wu Z., Ding W., Xiao C., Zhang Y., Gao S., Gao Y., Cai W. (2020). SREBP1 siRNA enhance the docetaxel effect based on a bone-cancer dual-targeting biomimetic nanosystem against bone metastatic castration-resistant prostate cancer. Theranostics.

[59] Li X., Chen Y.-T., Hu P., Huang W.-C. (2014). Fatostatin displays high antitumor activity in prostate cancer by blocking SREBP-regulated metabolic pathways and androgen receptor signaling. Mol. Cancer Ther..

[60] Chen M., Zhang J., Sampieri K., Clohessy J.G., Mendez L., Gonzalez-Billalabeitia E., Liu X.-S., Lee Y.-R., Fung J., Katon J.M. (2018). An aberrant SREBP-dependent lipogenic program promotes metastatic prostate cancer. Nat. Genet..

[61] Wall C.T.J., Lefebvre G., Metairon S., Descombes P., Wiederkehr A., Santo-Domingo J. (2022). Mitochondrial respiratory chain dysfunction alters ER sterol sensing and mevalonate pathway activity. J. Biol. Chem..

[62] Toshima T., Yagi M., Do Y., Hirai H., Kunisaki Y., Kang D., Uchiumi T. (2024). Mitochondrial translation failure represses cholesterol gene expression via Pyk2-Gsk3β-Srebp2 axis. Life Sci. Alliance.

[63] Ruiz C.F., Montal E.D., Haley J.A., Bott A.J., Haley J.D. (2020). SREBP1 regulates mitochondrial metabolism in oncogenic KRAS expressing NSCLC. FASEB J..

[64] He Y., Qi S., Chen L., Zhu J., Liang L., Chen X., Zhang H., Zhuo L., Zhao S., Liu S. (2024). The roles and mechanisms of SREBP1 in cancer development and drug response. Genes Dis..

[65] Bajgelman M.C., Strauss B.E. (2006). The DU145 human prostate carcinoma cell line harbors a temperature-sensitive allele of p53. Prostate.

[66] Seim I., Jeffery P.L., Thomas P.B., Nelson C.C., Chopin L.K. (2017). Whole-Genome Sequence of the Metastatic PC3 and LNCaP Human Prostate Cancer Cell Lines. G3 Genes Genomes Genet..

[67] Fraser M., Zhao H., Luoto K.R., Lundin C., Coackley C., Chan N., Joshua A.M., Bismar T.A., Evans A., Helleday T. (2012). PTEN Deletion in Prostate Cancer Cells Does Not Associate with Loss of RAD51 Function: Implications for Radiotherapy and Chemotherapy. Clin. Cancer Res..

[68] Allen J.E., Krigsfeld G., Patel L., Mayes P.A., Dicker D.T., Wu G.S., El-Deiry W.S. (2015). Identification of TRAIL-inducing compounds highlights small molecule ONC201/TIC10 as a unique anti-cancer agent that activates the TRAIL pathway. Mol. Cancer.

[69] Stein M.N., Bertino J.R., Kaufman H.L., Mayer T., Moss R., Silk A., Chan N., Malhotra J., Rodriguez L., Aisner J. (2017). First-in-Human Clinical Trial of Oral ONC201 in Patients with Refractory Solid Tumors. Clin. Cancer Res..

[70] Gardner S.L., Tarapore R.S., Allen J., McGovern S.L., Zaky W., Odia Y., Daghistani D., Diaz Z., Hall M.D., Khatib Z. (2022). Phase I dose escalation and expansion trial of single agent ONC201 in pediatric diffuse midline gliomas following radiotherapy. Neuro-Oncol. Adv..

[71] Prabhu V., Kawakibi A.R., Madhukar N., Garnett M., McDermott U., Benes C., Wechsler-Reya R., Elemento O., Stogniew M., Oster W. (2019). EXTH-71. Ind-enabling characterization of ONC206 as the next bitopic DRD2 antagonist for neuro-oncology. Neuro-Oncol..

[72] Allen J.E., Crowder R., El-Deiry W.S. (2015). First-In-Class Small Molecule ONC201 Induces DR5 and Cell Death in Tumor but Not Normal Cells to Provide a Wide Therapeutic Index as an Anti-Cancer Agent. PLoS ONE.

[73] Orellana E.A., Kasinski A.L. (2016). Sulforhodamine B (SRB) Assay in Cell Culture to Investigate Cell Proliferation. Bio Protoc..

[74] Grada A., Otero-Vinas M., Prieto-Castrillo F., Obagi Z., Falanga V. (2017). Research Techniques Made Simple: Analysis of Collective Cell Migration Using the Wound Healing Assay. J. Investig. Dermatol..

[75] Bahmad H.F., Cheaito K., Chalhoub R.M., Hadadeh O., Monzer A., Ballout F., El-Hajj A., Mukherji D., Liu Y.-N., Daoud G. (2018). Sphere-formation assay: Three-dimensional in vitro culturing of prostate cancer stem/progenitor sphere-forming cells. Front. Oncol..

[76] Andrews S., Krueger F., Segonds-Pichon A., Biggins L., Krueger C., Wingett S. (2010). FastQC. A Quality Control Tool for High Throughput Sequence Data.

[77] Wingett S.W., Andrews S. (2018). FastQ Screen: A tool for multi-genome mapping and quality control. F1000Research.

[78] Kim D., Paggi J.M., Park C., Bennett C., Salzberg S.L. (2019). Graph-based genome alignment and genotyping with HISAT2 and HISAT-genotype. Nat. Biotechnol..

[79] Danecek P., Bonfield J.K., Liddle J., Marshall J., Ohan V., Pollard M.O., Whitwham A., Keane T., McCarthy S.A., Davies R.M. (2021). Twelve years of SAMtools and BCFtools. Gigascience.

[80] Liao Y., Smyth G.K., Shi W. (2014). featureCounts: An efficient general purpose program for assigning sequence reads to genomic features. Bioinformatics.

[81] Love M.I., Huber W., Anders S. (2014). Moderated estimation of fold change and dispersion for RNA-seq data with DESeq2. Genome Biol..

[82] Kuleshov M.V., Jones M.R., Rouillard A.D., Fernandez N.F., Duan Q., Wang Z., Koplev S., Jenkins S.L., Jagodnik K.M., Lachmann A. (2016). Enrichr: A comprehensive gene set enrichment analysis web server 2016 update. Nucleic Acids Res..

[83] Gu Z., Eils R., Schlesner M. (2016). Complex heatmaps reveal patterns and correlations in multidimensional genomic data. Bioinformatics.

[84] Wickham H. (2016). Getting Started with ggplot2. ggplot2: Elegant Graphics for Data Analysis.

[85] Larsson J. (2021). eulerr: Area-Proportional Euler and Venn Diagrams with Ellipses.

