# Supplementary material for: Integrated Phenotypic and Transcriptomic Profiling Positions ONC212 as a Lead Imipridone in Androgen-Independent Prostate Cancer Models
Source: Int J Mol Sci. 2026 May 20;27(10):4597. doi: 10.3390/ijms27104597 (PMC13207072; doi:10.3390/ijms27104597)
Supplement: Supplementary file 1 [file ijms-27-04597-s001.zip › Supplementary Figures.pdf]

International Journal of
Molecular Sciences

MDPI

Article

Integrated Phenotypic and Transcriptomic Profiling Positions ONC212 as a Lead Imipridone in Androgen-Independent Prostate Cancer Models

Fatima Ghamlouche [1], Amani Yehya [1], Abdallah Kurdi [2], Sana Hachem [1], Varun V. Prabhu [3], Georges Daoud [1,†] and Wassim Abou-Kheir [1,*,†]

[1] Department of Anatomy, Cell Biology and Physiological Sciences, Faculty of Medicine, American University of Beirut, Beirut P.O. Box 11-0236, Lebanon
[2] Department of Biochemistry and Molecular Genetics, Faculty of Medicine, American University of Beirut, Beirut P.O. Box 11-0236, Lebanon
[3] Chimerix, Inc., Durham, NC 27713, USA
* Correspondence: wa12@aub.edu.lb
† These authors contributed equally to this work.

Supplementary Figures

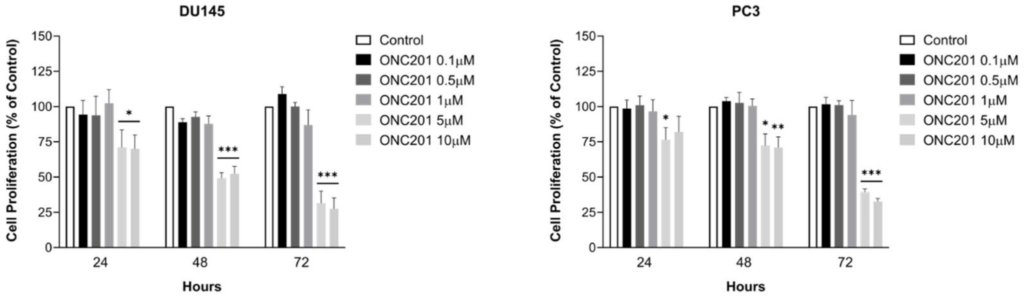

Supplementary Figure S1. ONC201 reduced the proliferation of human androgen-independent prostate cancer cells in a time- and dose-dependent manner. DU145 and PC3 cells were treated for 24, 48, or 72 h with the indicated concentrations of ONC201, and cell proliferation was measured by thiazolyl blue tetrazolium bromide (MTT) assay. Results are normalized to the vehicle control at each time point and shown as mean ± SEM from ≥ 3 independent experiments. Statistical significance was assessed by two-way ANOVA with Tukey's post hoc multiple-comparisons test; *$p < 0.05$, **$p < 0.01$, ***$p < 0.001$.

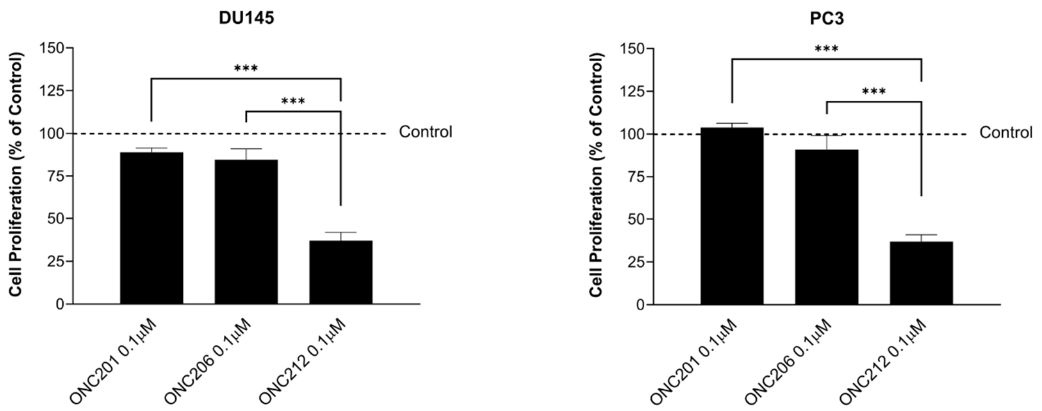

Supplementary Figure S2. ONC212 showed greater antiproliferative activity than ONC201 or ONC206 at 0.1 µM after 48 h in human androgen-independent prostate cancer cells. DU145 and PC3 cells were treated for 48 h with 0.1 µM of ONC201, ONC206, or ONC212. Cell proliferation was measured by thiazolyl blue tetrazolium bromide (MTT) and normalized to the vehicle control (dashed line = 100%). Bars show mean ± SEM from ≥ 3 independent experiments. Statistical significance was assessed by one-way ANOVA with Tukey's post hoc multiple-comparisons test; ***$p < 0.001$. Data corresponds to the 48 h time point, 0.1 µM condition from the experiment presented in Figure 2A, 2B and Supplementary Figure S1.

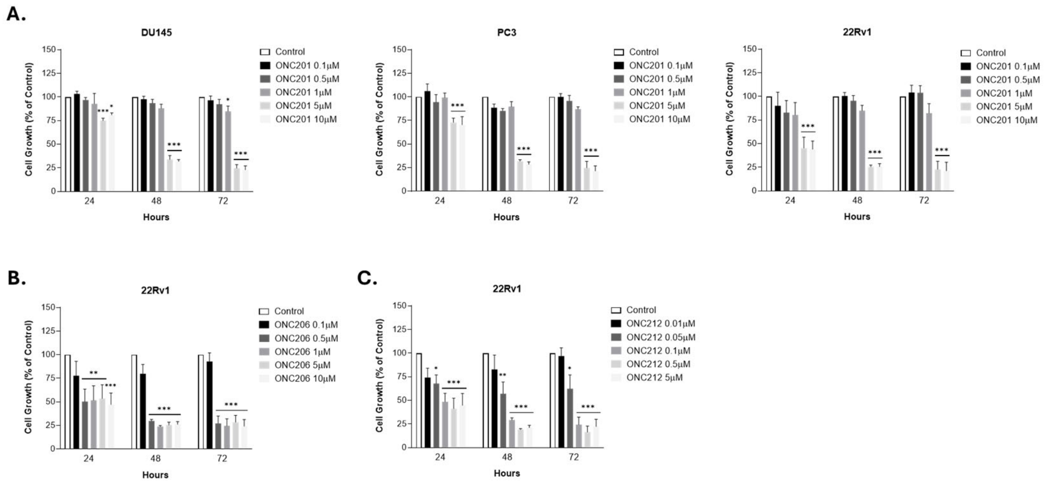

Supplementary Figure S3. ONC201, ONC206, or ONC212 reduced the growth of human androgen-independent prostate cancer cells in a time- and dose-dependent manner. Androgen-independent prostate cancer cells were treated for 24, 48, or 72 h with the indicated concentrations of ONC201 (A), ONC206 (B), or ONC212 (C) and cell growth was measured by Sulforhodamine B (SRB) assay. Results are normalized to the vehicle control at each time point and shown as mean ± SEM from ≥ 3 independent experiments. Statistical significance was assessed by two-way ANOVA with Dunnett's post hoc multiple-comparisons test; *$p < 0.05$, **$p < 0.01$, ***$p < 0.001$.

https://doi.org/10.3390/ijms27104597

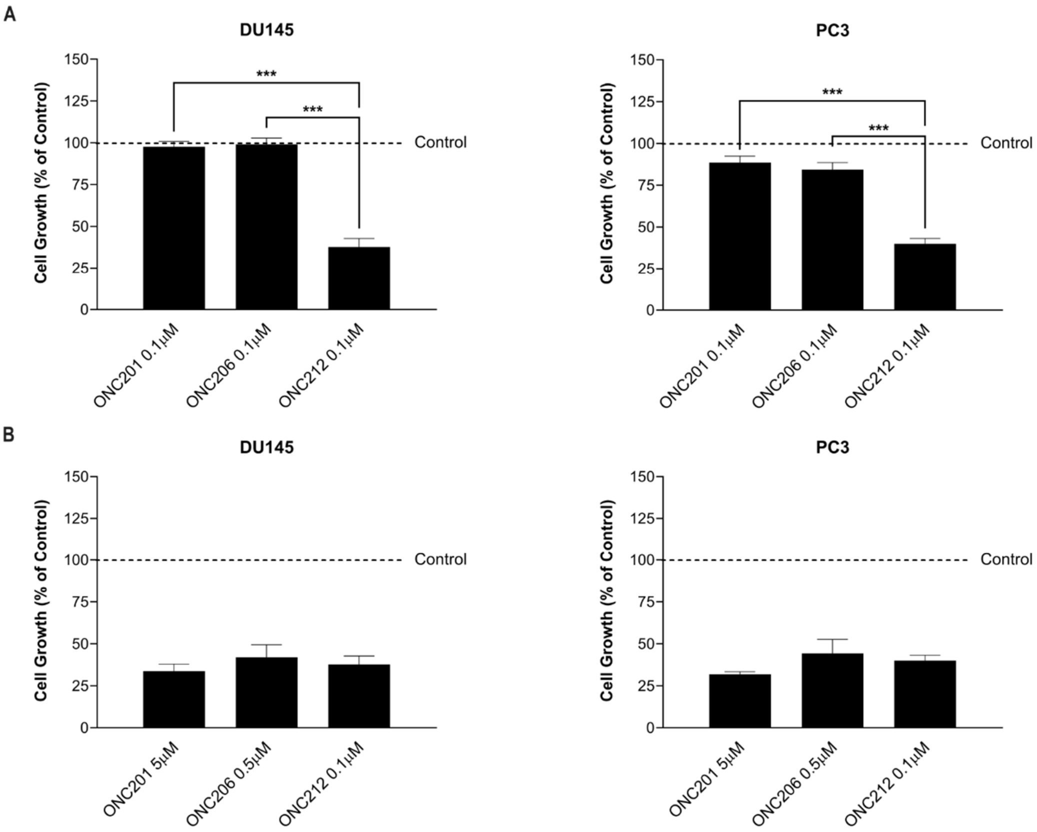

Supplementary Figure S4. ONC212 inhibited human androgen-independent prostate cancer cell growth more potently than ONC201 or ONC206 after 48 h. (A) DU145 and PC3 cells were treated for 48 h with 0.1 μM of ONC201, ONC206, or ONC212. (B) DU145 and PC3 cells were treated for 48 h with 5 μM of ONC201, 0.5 μM of ONC206, or 0.1 μM of ONC212. Cell growth was quantified by sulforhodamine B (SRB) assay and normalized to the vehicle control (dashed line = 100%). Bars show mean ± SEM from ≥ 3 independent experiments. Data corresponds to the 48 h time point from the experiment presented in Figure 2C, 2D and Supplementary Figure S3A.

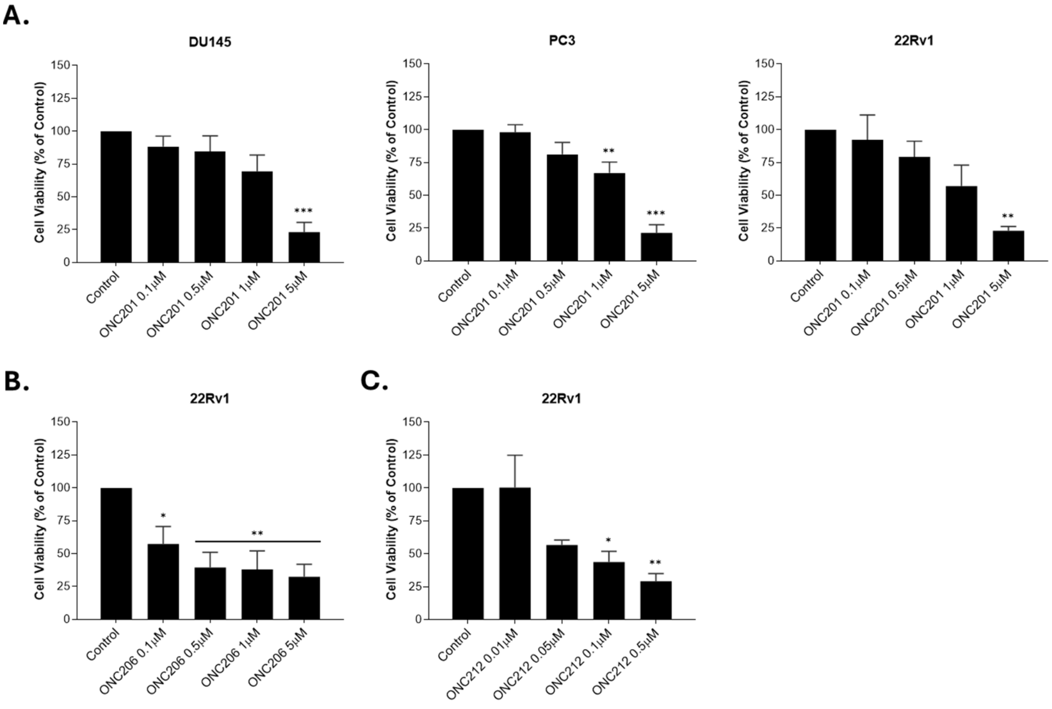

Supplementary Figure S5. ONC201, ONC206, or ONC212 reduced the viability of human androgen-independent prostate cancer cells. DU145, PC3, and 22Rv1 cells were treated for 48 h with the indicated concentrations of ONC201 (A), ONC206 (B), or ONC212 (C) and cell viability was measured by trypan blue exclusion assay. Results are normalized to vehicle control and shown as mean ± SEM from ≥ 3 independent experiments. Statistical significance was assessed by one-way ANOVA with Dunnett's post hoc multiple-comparisons test; ***p* < 0.01, ****p* < 0.001.

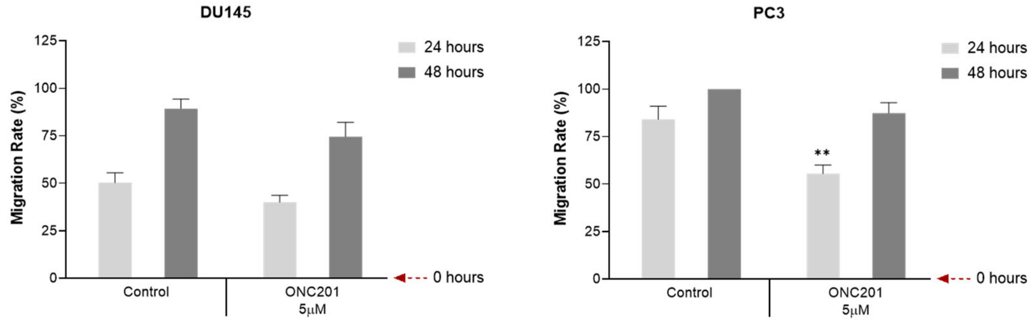

Supplementary Figure S6. ONC201 reduced the migratory capacity of PC3 cells after 24 h. Confluent monolayers of DU145 and PC3 cells were pretreated with mitomycin C to inhibit proliferation, scratched with a sterile 200 μL pipette tip, and treated with ONC201 (5 μM); vehicle served as control. Images were acquired at 0, 24, and 48 h (5× magnification). Wound area was quantified using ImageJ, and migration rate (%) was calculated and presented. Bars show mean ± SEM from ≥ 4 independent experiments. Statistical significance was assessed by two-way ANOVA with Dunnett's post hoc test vs time-matched control; ***p* < 0.01.

https://doi.org/10.3390/ijms27104597

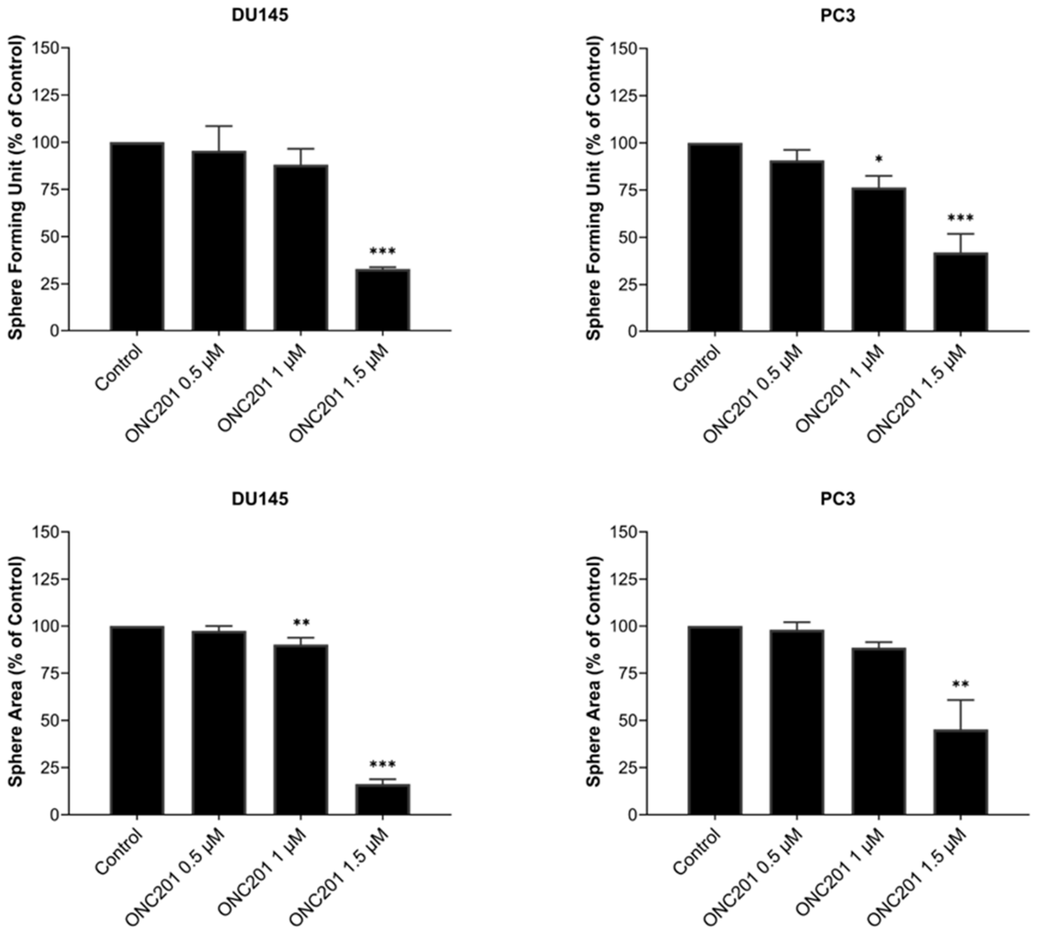

Supplementary Figure S7. ONC201 reduced the formation and growth of human prostate cancer spheroids in three-dimensional culture. DU145 and PC3 cells were embedded in Matrigel™ and cultured with the indicated concentrations of ONC201, replenished every other day. Spheres were counted 7–8 days post-seeding and imaged using an Axiovert light microscope at 10× magnification. Number of spheres counted is expressed as sphere-forming unit (SFU %), normalized to the vehicle control. The mean sphere area (μm^2) was determined from ≥ 30 spheres per condition. Data represents the mean ± SEM of ≥ 3 independent experiments. Statistical significance was assessed by one-way ANOVA with Holm-Šídák's post hoc multiple-comparisons test; *$p < 0.05$, **$p < 0.01$, ***$p < 0.001$.

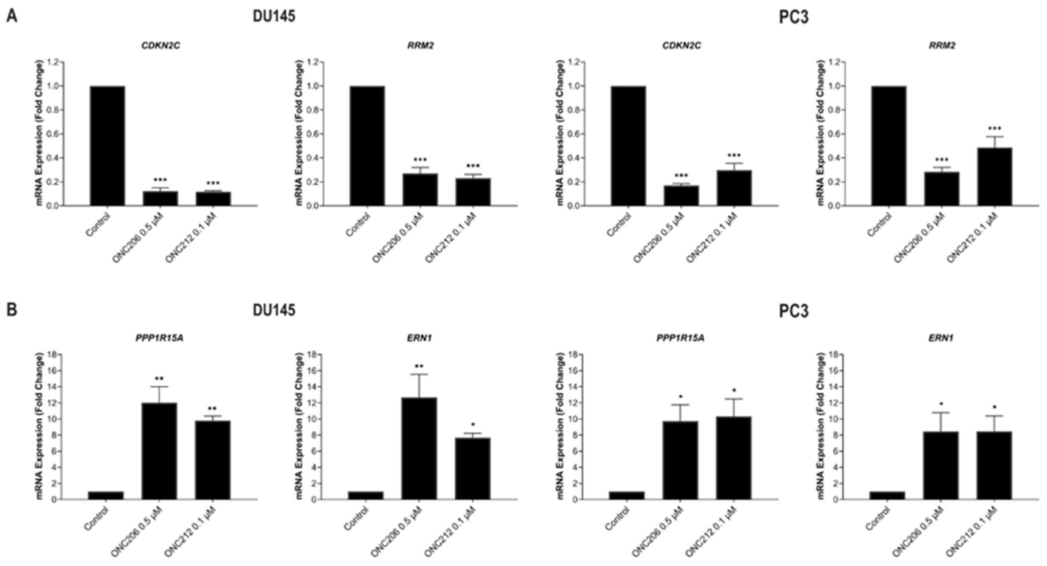

Supplementary Figure S8. Quantitative real-time PCR confirmed RNA-sequencing-identified expression changes in human androgen-independent prostate cancer cells treated with imipridones derivatives. Gene expression levels of selected downregulated (A) and upregulated (B) targets identified from the RNA-sequencing analysis were quantified by reverse transcription quantitative real-time PCR (qRT-PCR). DU145 and PC3 cells were treated with ONC206 (0.5 μM) or ONC212 (0.1 μM) for 48 h. Total RNA was reverse-transcribed and amplified using SYBR Green PCR Master Mix. Expression levels were normalized to *ACTB* and *GAPDH* and calculated using the comparative Ct method (ΔΔCt). Bars show mean ± SEM from 3 independent experiments. Statistical significance was assessed by one-way ANOVA with Holm-Šídák's post hoc multiple-comparisons test; *$p < 0.05$, **$p < 0.01$, ***$p < 0.001$.

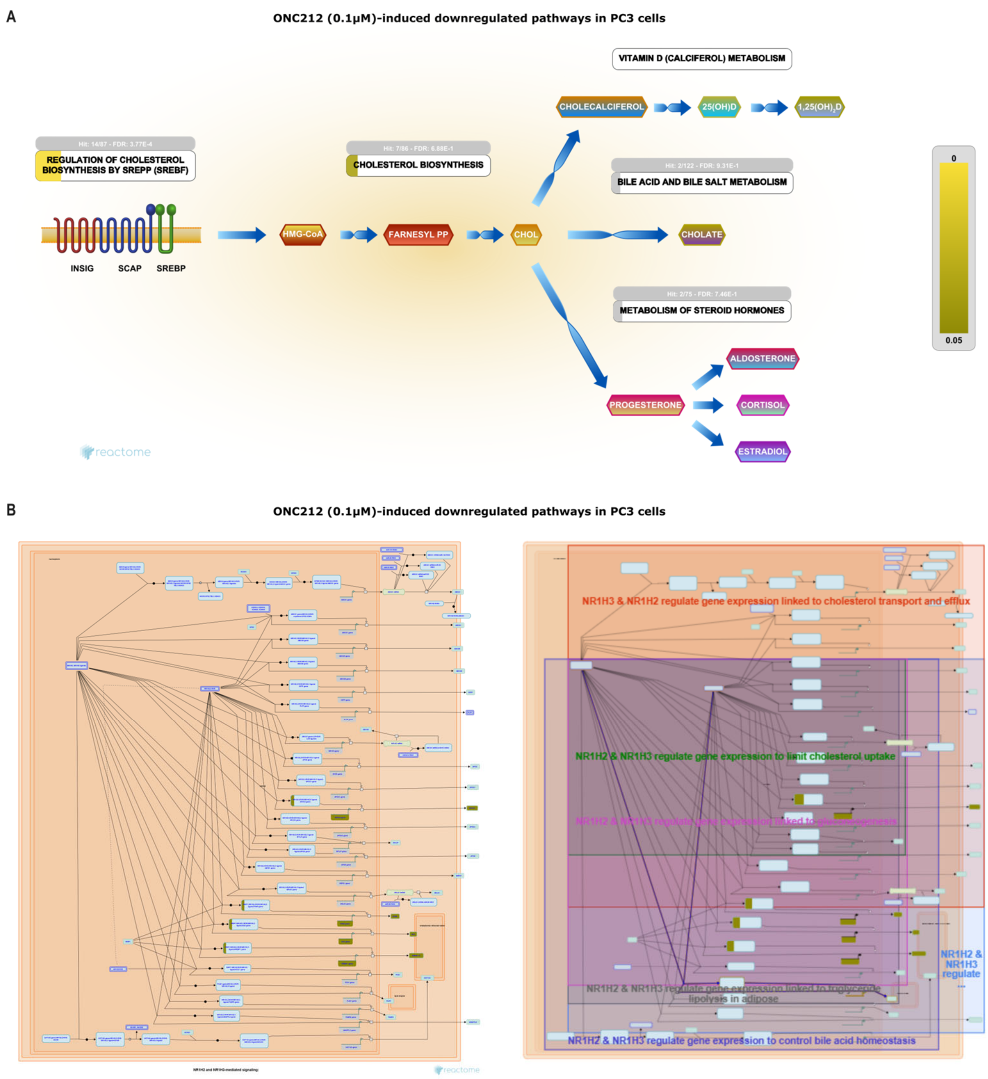

Supplementary Figure S9. Reactome pathways suggested downregulation of lipogenic and cholesterol biosynthesis signaling in PC3 cells treated with ONC212. (A) Representative Reactome overlay maps (downregulated transcript sets) are shown for PC3 cells treated with ONC212 (0.1 μM), demonstrating suppression of steroids' metabolism modules, including regulation of cholesterol biosynthesis by SREBF (SREBP), Cholesterol biosynthesis, bile acid and bile salt metabolism, and metabolism of steroid hormones. (B) Two Reactome overlays maps representing the same pathway, NR1H2 & NR1H3-mediated signaling, linked to lipogenesis. Left, compact detailed network view; Right, decomposed/annotated view highlighting constituent submodules. Color bars indicate Reactome overlay significance; full statistics are provided in Supplementary Table S4.

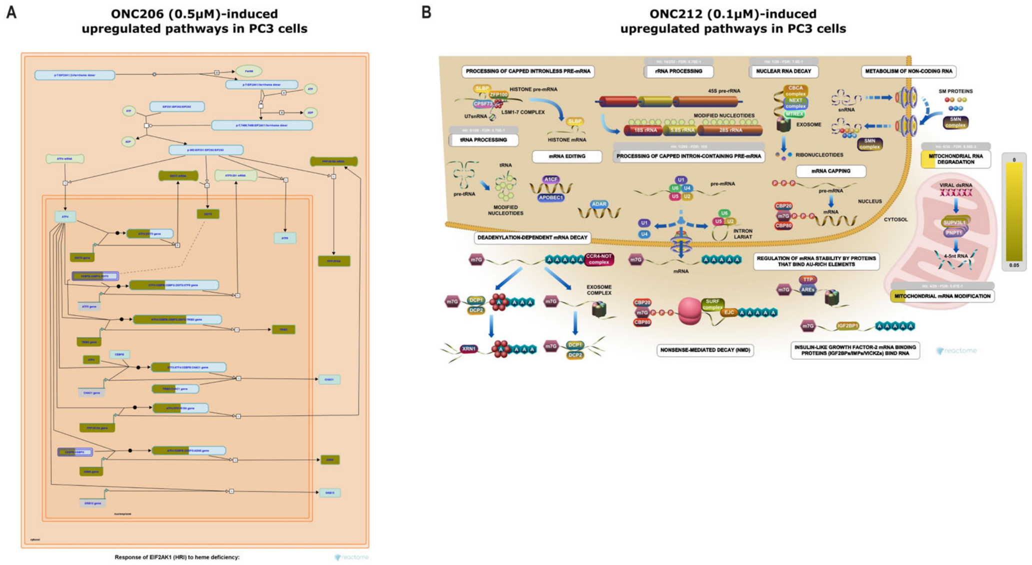

Supplementary Figure S10. Upregulated Reactome pathways showed PERK-regulated unfolded protein response and mitochondrial RNA-metabolism in PC3 cells treated with ONC206 and ONC212, respectively. (A) Representative Reactome overlay maps (upregulated transcript sets) are shown for PC3 cells treated with ONC206 (0.5 μM), demonstrating stronger PERK (EIF2AK3)–regulated gene expression within the unfolded protein response (UPR). (B) Representative Reactome overlay maps (upregulated transcript sets) are shown for PC3 cells treated with ONC212 (0.1 μM), indicating preferential enrichment of mitochondrial RNA-metabolism (tRNA processing and rRNA processing). Color bars indicate Reactome overlay significance; full statistics are provided in Supplementary Table S5.

https://doi.org/10.3390/ijms27104597

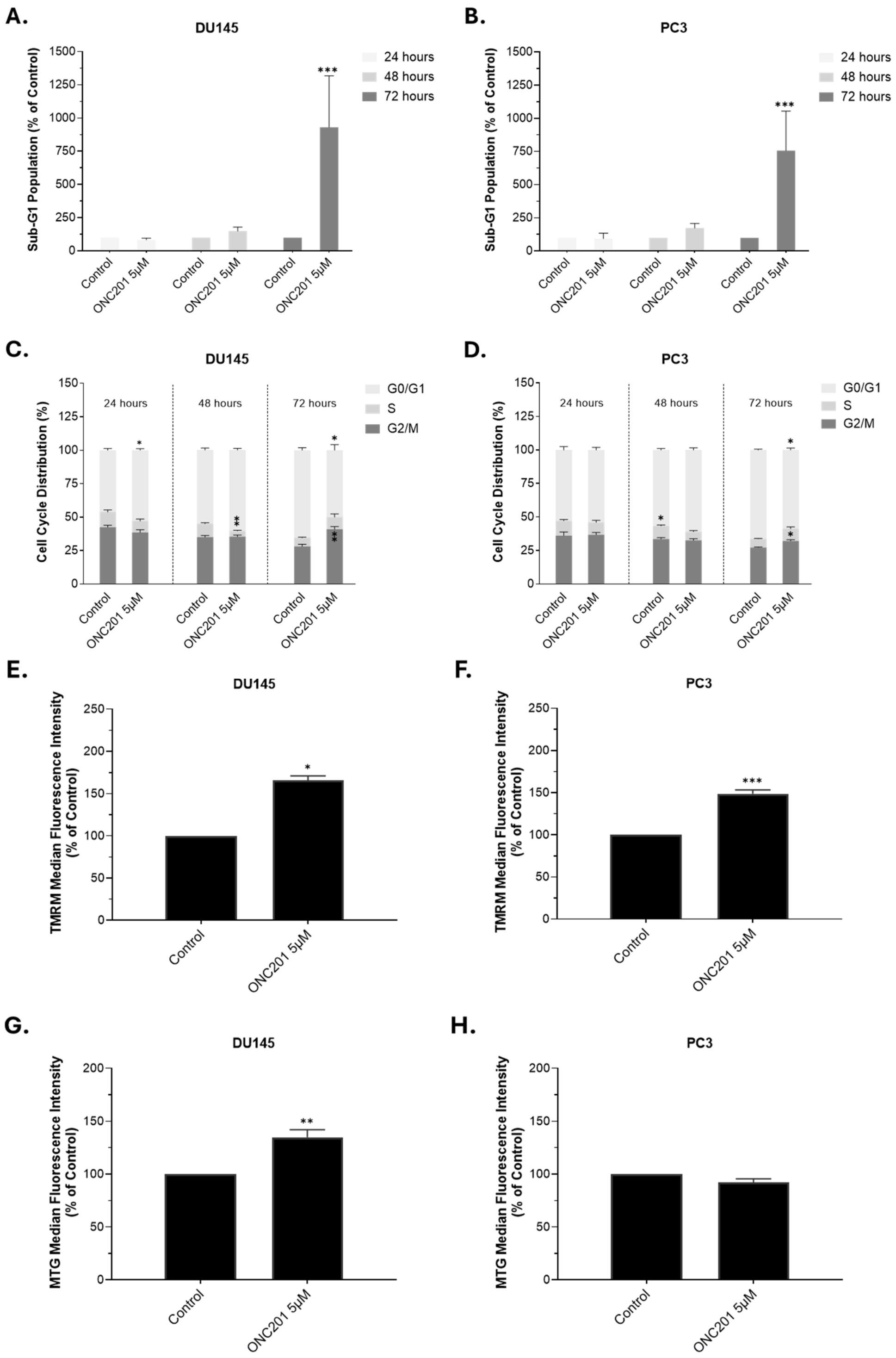

Supplementary Figure S11. ONC201 induced cell-cycle redistribution, Sub-G1 accumulation, and modulation of mitochondrial membrane potential and mass in androgen-independent prostate cancer cells. (A-D) DU145 and PC3 cells were treated with ONC201 (5 μM) for 24, 48, or 72 h. Cell-cycle distribution was analyzed by propidium iodide (PI) staining and flow cytometry. (A,B) Bars show quantification of sub-G1 fraction is shown, normalized to vehicle control at each time point. (C,D) Bars show distribution of cells across G0/G1, S, and G2/M phases. Data represents the mean ± SEM of ≥ 4 independent experiments. (E-G) DU145 and PC3 cells were treated with ONC201 (5 μM) for 48 h. (E,F) Mitochondrial membrane potential was assessed by Tetramethylrhodamine, Methyl Ester, Perchlorate (TMRM) staining and flow cytometry. (G,H) Mitochondrial mass was evaluated using MitoTracker Green (MTG). Median fluorescence intensity (MFI) was normalized to vehicle control. Data Represents the mean ± SEM of ≥ 3 independent experiments. Statistical significance was determined by one- or two-way ANOVA with appropriate multiple-comparisons tests; **p* < 0.05, ***p* < 0.01, ****p* < 0.001.

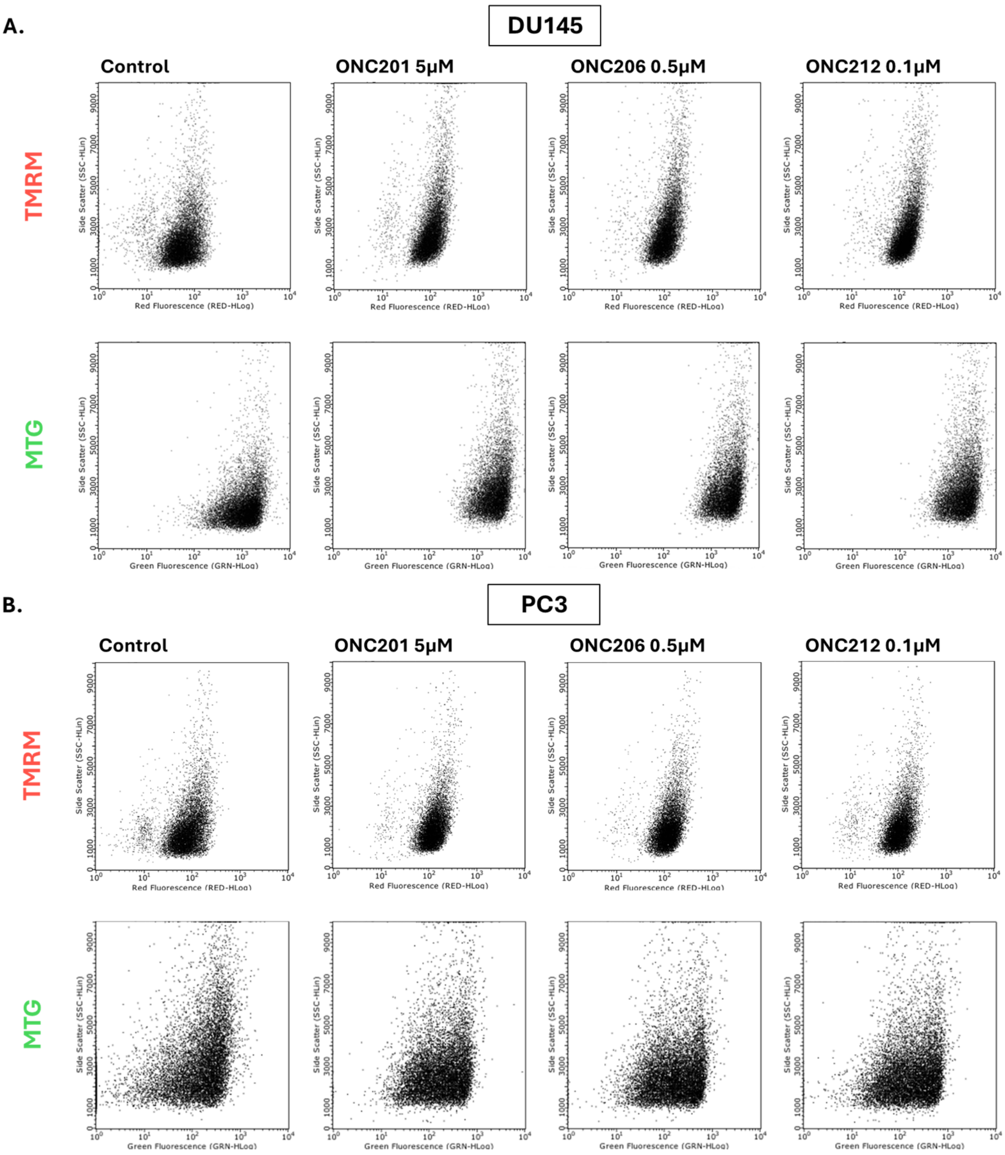

C.

DU145		Control	ONC201 5μM	ONC206 0.5μM	ONC212 0.1μM
Average MFI	TMRM	100.0	165.9	160.7	179.3
	MTG	100.0	134.8	126.7	134.0
TMRM Normalized to MTG	TMRM / MTG	1.00	1.23	1.27	1.34

D.

PC3		Control	ONC201 5μM	ONC206 0.5μM	ONC212 0.1μM
Average MFI	TMRM	100.0	148.7	142.8	130.0
	MTG	100.0	92.1	89.5	92.6
TMRM Normalized to MTG	TMRM / MTG	1.00	1.61	1.59	1.40

Supplementary Figure S12. ONC201, ONC206, or ONC212 modulated mitochondrial membrane potential and mitochondrial mass in androgen-independent prostate cancer cells. (A,B) Representative flow cytometry bivariate scatter plots of Tetramethylrhodamine, Methyl Ester, Perchlorate (TMRM) staining (red-orange, fluorescent dye for mitochondrial membrane potential) and MitoTracker Green (MTG; green, fluorescent dye for mitochondrial mass) in DU145 (A) and PC3 (B) cells 48 h post-treatment with ONC201 (5 μM), ONC206 (0.5 μM), or ONC212 (0.1 μM). (C,D) Quantification of average median fluorescence intensity (MFI) values for TMRM and MTG in DU145 (C) and PC3 (D) cells 48 h post-treatment with the imipridones. MFI values are expressed as percentages relative to DMSO-treated control (set to 100). TMRM signals were normalized to MTG intensity (TMRM/MTG ratio). Control normalized ratio was set to 1.00.

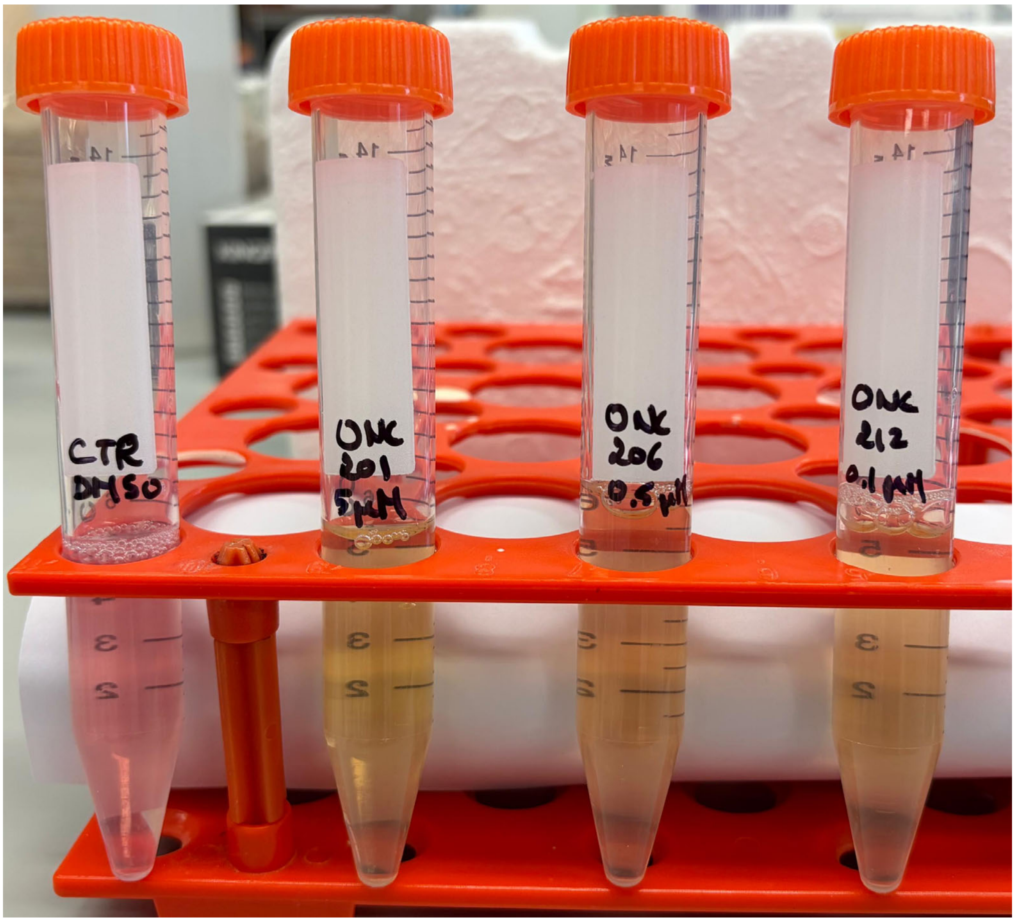

Supplementary Figure S13. ONC201, ONC206, or ONC212 were associated with a visible phenol red color shift consistent with extracellular acidification. Representative image of phenol red–containing culture medium collected from PC3 prostate cancer cell line following treatment with DMSO vehicle-treated control (CTR DMSO), ONC201 (5 μM), ONC206 (0.5 μM), or ONC212 (0.1 μM) for 48 h. Imipridone-treated conditions show a shift from pink/red toward orange/yellow relative to control, consistent with reduced extracellular pH.

https://doi.org/10.3390/ijms27104597
