# Supplementary material for: Integrated Phenotypic and Transcriptomic Profiling Positions ONC212 as a Lead Imipridone in Androgen-Independent Prostate Cancer Models
Source: Int J Mol Sci. 2026 May 20;27(10):4597. doi: 10.3390/ijms27104597 (PMC13207072; doi:10.3390/ijms27104597)
Supplement: Supplementary file 1 [file ijms-27-04597-s001.zip › Supplementary Table S1.pdf]

International Journal of
Molecular Sciences

MDPI

Article

Integrated Phenotypic and Transcriptomic Profiling Positions ONC212 as a Lead Imipridone in Androgen-Independent Prostate Cancer Models

Fatima Ghamlouche [1], Amani Yehya [1], Abdallah Kurdi [2], Sana Hachem [1], Varun V. Prabhu [3], Georges Daoud [1,†] and Wassim Abou-Kheir [1,*,†]

[1] Department of Anatomy, Cell Biology and Physiological Sciences, Faculty of Medicine, American University of Beirut, Beirut P.O. Box 11-0236, Lebanon
[2] Department of Biochemistry and Molecular Genetics, Faculty of Medicine, American University of Beirut, Beirut P.O. Box 11-0236, Lebanon
[3] Chimerix, Inc., Durham, NC 27713, USA
* Correspondence: wa12@aub.edu.lb
† These authors contributed equally to this work.

Supplementary Tables

Supplementary Table S1. Growth Inhibitory Concentration 50% (GI_{50}) values for ONC201, ONC206, and ONC212 in DU145 and PC3 cells. GI_{50} values (μM) at 24, 48, and 72 h were calculated by nonlinear regression from sulforhodamine B (SRB) dose–response curves following continuous exposure to ONC201 (0.1–10 μM), ONC206 (0.1–10 μM), or ONC212 (0.05–5 μM).

**Cell Line**	**DU145**			**PC3**		
Time Point (hours)	**24**	**48**	**72**	**24**	**48**	**72**
GI_{50} of ONC201	>10 †	3.89	2.92	>10 †	3.45	3.05
GI_{50} of ONC206	>10 †	0.65	0.26	>10 †	0.68	0.40
GI_{50} of ONC212	>5 †	0.11	0.08	>5 †	0.13	0.10

† GI_{50} was not reached within the tested concentration range and is therefore reported as greater than the maximum tested concentration (ONC201/ONC206: 10 μM; ONC212: 5 μM).

 https://doi.org/10.3390/ijms27104597
